# CRISPR-Cas9-mediated knockout of *CYP79D1* and *CYP79D2* in cassava attenuates toxic cyanogen production

**DOI:** 10.3389/fpls.2022.1079254

**Published:** 2023-03-17

**Authors:** Michael A. Gomez, Kodiak C. Berkoff, Baljeet K. Gill, Anthony T. Iavarone, Samantha E. Lieberman, Jessica M. Ma, Alex Schultink, Nicholas G. Karavolias, Stacia K. Wyman, Raj Deepika Chauhan, Nigel J. Taylor, Brian J. Staskawicz, Myeong-Je Cho, Daniel S. Rokhsar, Jessica B. Lyons

**Affiliations:** ^1^ Innovative Genomics Institute, University of California, Berkeley, Berkeley, CA, United States; ^2^ Department of Molecular & Cell Biology, University of California, Berkeley, Berkeley, CA, United States; ^3^ California Institute for Quantitative Biosciences (QB3), University of California, Berkeley, Berkeley, CA, United States; ^4^ Department of Plant & Microbial Biology, University of California, Berkeley, Berkeley, CA, United States; ^5^ Donald Danforth Plant Science Center, St. Louis, MO, United States; ^6^ US Department of Energy Joint Genome Institute, Lawrence Berkeley National Laboratory, Berkeley, CA, United States; ^7^ Molecular Genetics Unit, Okinawa Institute of Science and Technology Graduate University, Onna, Okinawa, Japan; ^8^ Chan-Zuckerberg BioHub, San Francisco, CA, United States

**Keywords:** cassava (*Manihot esculenta* Crantz), cyanide, cyanogenesis, CRISPR (Clustered Regularly Interspaced Short Palindromic Repeats)-Cas9 (CRISPR-associated protein 9), genome editing, CYP79D, climate resilience, food safety

## Abstract

Cassava (*Manihot esculenta*) is a starchy root crop that supports over a billion people in tropical and subtropical regions of the world. This staple, however, produces the neurotoxin cyanide and requires processing for safe consumption. Excessive consumption of insufficiently processed cassava, in combination with protein-poor diets, can have neurodegenerative impacts. This problem is further exacerbated by drought conditions which increase this toxin in the plant. To reduce cyanide levels in cassava, we used CRISPR-mediated mutagenesis to disrupt the cytochrome P450 genes *CYP79D1* and *CYP79D2* whose protein products catalyze the first step in cyanogenic glucoside biosynthesis. Knockout of both genes eliminated cyanide in leaves and storage roots of cassava accession 60444; the West African, farmer-preferred cultivar TME 419; and the improved variety TMS 91/02324. Although knockout of *CYP79D2* alone resulted in significant reduction of cyanide, mutagenesis of *CYP79D1* did not, indicating these paralogs have diverged in their function. The congruence of results across accessions indicates that our approach could readily be extended to other preferred or improved cultivars. This work demonstrates cassava genome editing for enhanced food safety and reduced processing burden, against the backdrop of a changing climate.

## 1 Introduction

The starchy root crop cassava (*Manihot esculenta* Crantz, also known as tapioca, yuca, or manioc) is an important staple for over a billion people in tropical and subtropical regions of the world, including roughly 40% of Africans (Nweke, 2004; Lebot, 2019). It is an excellent food security crop due to its tolerance for drought and marginal soils, and because its tuberous roots can remain in the ground until needed (Howeler et al., 2013). A major challenge, however, is the presence of toxic cyanogenic compounds (e.g., cyanogenic glucosides) in cassava, which must be removed by post-harvest processing to prevent cyanide exposure and illness. Cassava root processing can be laborious, results in nutrient loss, and in Africa falls disproportionately on women and girls (Chiwona‐Karltun et al., 1998; Curran et al., 2009; Maziya-Dixon et al., 2009; Montagnac et al., 2009; Boakye Peprah et al., 2020). Troublingly, cyanogen levels in cassava increase under drought stress (El-Sharkawy, 1993; Okogbenin et al., 2003; Vandegeer et al., 2013; Brown et al., 2016). As drought frequency, duration, and severity are projected to increase due to climate change (Ayugi et al., 2022), cassava consumers’ risk of cyanide exposure may increase as well.

Following cellular disruption (e.g., during ingestion), cyanogenic glucosides are broken down to release the toxin cyanide. Distributed throughout the body *via* the bloodstream, cyanide halts mitochondrial electron transport, thereby preventing cells from using oxygen to produce energy and causing cell death (Dobbs, 2009). The central nervous system is particularly impacted by this toxin due to its substantial oxygen demand. The risks of insufficient cassava processing include acute cyanide poisoning which can be fatal. Chronic cyanide exposure from dietary intake induces the paralytic disease konzo, is associated with neurodevelopmental deficits, and exacerbates tropical ataxic neuropathy and goiter (Nhassico et al., 2008; Nzwalo and Cliff, 2011; Tshala-Katumbay et al., 2016; Kashala-Abotnes et al., 2018). Sulfur-containing amino acids are required to detoxify cyanide in the body; thus, those with a protein-poor diet heavily reliant on cassava are particularly at risk for adverse effects from cyanide exposure (Nzwalo and Cliff, 2011). Konzo is more likely to occur in women of childbearing age and children (Baguma et al., 2021).

Processing to remove cyanogenic content from tuberous roots can be achieved by chipping and air drying, grinding, mashing and steeping, and/or fermentation. All require 24 hours to several days to complete. Though premature consumption exposes consumers to risk, shortcuts are sometimes taken during processing, especially when food is in short supply (Banea et al., 1992; Essers et al., 1992; McKey et al., 2010; Fitzpatrick et al., 2021). Processing approaches vary by region and specific cultivated variety (cultivar) used. There are cultural preferences for growing high cyanogenic (known as “bitter”) cultivars in some contexts, for example to deter theft (Chiwona‐Karltun et al., 1998). A mismatch between expected and actual cyanide levels (due to use of a different cultivar or environmental factors) may render the usual processing insufficient. Industrial scale processing of cassava poses risks to the environment and to workers through cyanide release into wastewater and the air, respectively (Adewoye et al., 2005; Ehiagbonare et al., 2009; Dhas et al., 2011). Cyanide levels above WHO recommendations have been found in commercial cassava products as well as household flour (Burns et al., 2012; Kashala-Abotnes et al., 2018).

**Figure 1:**
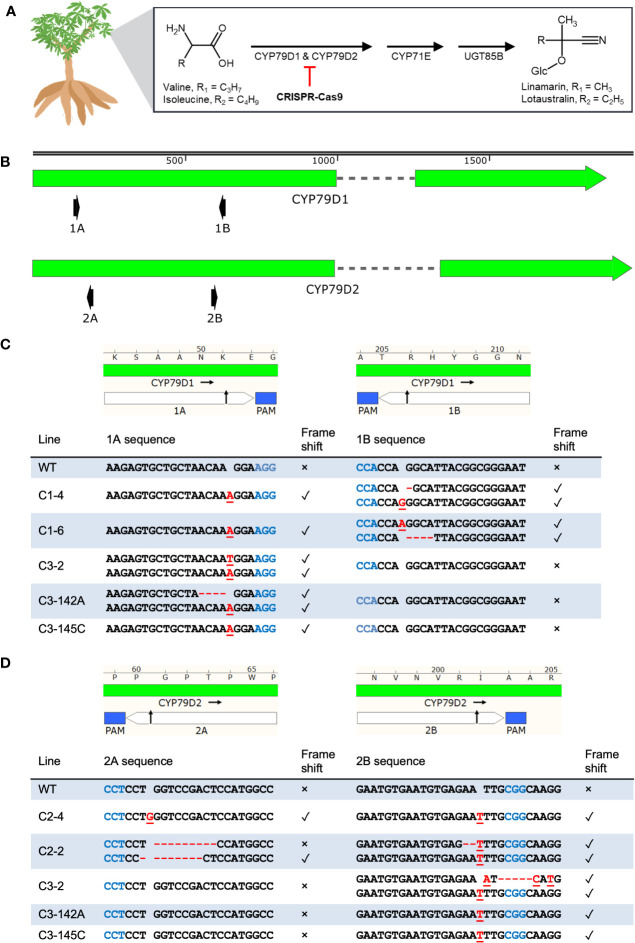
CRISPR-Cas9 induces indels at *CYP79D1* and *CYP79D2* gRNA target sites in transgenic 60444 lines. **(A)** Cassava biosynthetic pathway for the cyanogenic glucosides linamarin and lotaustralin. This process primarily occurs in the leaves. The step catalyzed by CYP79D1 and CYP79D2 enzymes was selected for disruption by the CRISPR-Cas9 system. Respective side chains are labeled as R1 and R2. Glc, glucose. Chemical structures created using ACD/Chemsketch (“ACD/Chemsketch, Version 2021.1.1” 2021). **(B)** Lengths of *CYP79D1* and *CYP79D2* genes are to nucleotide scale (top bar). Exons are denoted by solid blocks and introns are represented as dashed lines. Arrowheads indicate the 3′ terminus. Diagrams of the protospacers (white) and protospacer adjacent motifs (PAMs, blue) of *CYP79D1* **(C)** and *CYP79D2* **(D)** gRNA targets are aligned to edited line genotypes. Edited lines are identified by the CRISPR construct with which they were modified (C1, C2, C3), followed by an index number (e.g., 142A). Black arrow indicates predicted CRISPR-Cas9 cut site. Lengths are to amino acid (top bar) and nucleotide (bottom table) scale. Homozygous genotypes are shown as a single sequence per line. Bi-allelic genotypes are shown as two sequences per line. Mutations on the same haplotype at 1A and 1B sites in a given mutant line are shown in the same row. Insertions are denoted by red, underlined nucleotides. Deletions are denoted by red dashes. Presence of a frameshift mutation at the corresponding target site is denoted by ✓; absence of a frameshift mutation is denoted by ×. WT, wildtype. Maps created with SnapGene.

The biosynthetic pathway for cyanogenic glucosides requires cytochrome P450 (CYP) enzymes of the CYP79 family (Luck et al., 2016). In cassava, the enzymes CYP79D1 and CYP79D2 catalyze the first, limiting step of cyanogen biosynthesis (Andersen et al., 2000) (**Figure 1A**). The genes *CYP79D1* and *CYP79D2* are paralogous, having arisen through the whole-genome duplication found in this lineage (Bredeson et al., 2016). Cassava’s principal cyanogens, linamarin and lotaustralin, derived from valine and isoleucine, respectively, are synthesized in the canopy and transported to the storage roots (Nartey, 1968; Jørgensen et al., 2005). Linamarin accounts for greater than 90% of cassava cyanogens (Nartey, 1968).

Cyanogens play multiple roles in plants including defense and metabolism. Although cyanogens can deter herbivores (Bernays et al., 1977; Rajamma and Premkumar, 1994; Gleadow and Møller, 2014), this is not the case for cassava in all contexts or against all herbivores, possibly due to coevolution (Riis et al., 2003; Pinto-Zevallos et al., 2016). For example, the whitefly *Bemisia tabaci* detoxifies cyanogenic glucosides by enzymatic conversion to inert derivatives (Easson et al., 2021). It has been proposed that cyanogens shuttle reduced nitrogen to cassava roots for protein synthesis; increased nitrate reductase activity in roots, however, may compensate for reduced cyanogen availability (Siritunga and Sayre, 2004; Jørgensen et al., 2005; Narayanan et al., 2011; Zidenga et al., 2017). Cyanogens are also hypothesized to play a role in initiating postharvest physiological deterioration of the roots by triggering reactive oxygen species production (Zidenga et al., 2012). Modulation of cyanide levels may, therefore, bolster the longevity of harvested roots. Generation of acyanogenic cassava will facilitate further investigation of cyanogenic potential in these roles.

Cyanogen production varies naturally among cultivars (Whankaew et al., 2011; Ogbonna et al., 2021; Ospina et al., 2021). RNAi knockdown of the *CYP79D* genes reduced cyanogen levels in cassava; knockdown plants displayed wildtype morphology in soil (Siritunga and Sayre, 2003; Jørgensen et al., 2005; Piero, 2015). These observations indicate that cyanogen levels can be modulated without disrupting other desirable plant properties.

Here, we show that cassava cyanogenesis can be prevented *via* genome editing. We used CRISPR (Clustered Regularly Interspaced Short Palindromic Repeats)-Cas9 (CRISPR-associated protein 9) mutagenesis to knock out the *CYP79D* genes in the model variety 60444; the popular West African landrace TME 419; and the improved variety TMS 91/02324, which retains robust resistance to cassava mosaic disease following regeneration through somatic embryogenesis (Chauhan et al., 2018). *Agrobacterium*-mediated CRISPR-Cas9 editing is efficient in cassava (Odipio et al., 2017; Bull et al., 2018; Hummel et al., 2018; Gomez et al., 2019; Veley et al., 2021). In contrast to RNAi knockdown, our targeted genome editing approach provides a precise, complete, and permanent loss of function, not requiring the ongoing expression of a transgene. The efficiency and precision of CRISPR-Cas9 editing are advantageous in this vegetatively propagated crop for which conventional breeding is laborious. We find that dual knockouts eliminate cyanogenic potential in all three cassava accessions. Single gene knockout lines reveal differential contribution of the two *CYP79D* genes to cassava cyanogenesis. The knockout lines described here facilitate further research into the role of cyanogens in cassava, and chart a course toward the development of acyanogenic planting materials.

## 2 Results

We disabled *CYP79D1* and *CYP79D2* using CRISPR-Cas9 constructs with guide RNAs (gRNAs) targeting the two genes, both singly and in combination (**Table 1**; **Figure 1B**; **Supplementary Figure 1**, Methods). For each gene, we selected two gRNAs with minimal off-target potential that were ~500 bp apart. We assembled these gRNAs into CRISPR constructs, and confirmed construct functionality *in planta* by adapting a geminivirus system in the surrogate model *Nicotiana benthamiana* (Baltes et al., 2014) (**Supplementary Note 1**; **Supplementary Figure 2**, Methods). These constructs were transformed using *Agrobacterium* into friable embryogenic calli (FEC) from the three cassava accessions. For each construct-accession pair, we recovered multiple independent T0 transgenic plant lines and characterized the induced mutations using Sanger sequencing (**Figures 1C, D**; **Supplementary Figures 3**, **4**; **Supplementary Data File 1**). We found mutagenesis of targets 1A and 2B occurred at a higher frequency than of 1B and 2A, which may be due to differences in the gRNA sequences and their respective binding efficiencies. Rarely was the region between the two target sites within a gene deleted. This result was unexpected since this excision, by all CRISPR constructs, was easily detectable in the *N. benthamiana* surrogate assay. Simultaneous cleavage of the target sites and excision may have occurred more frequently in this assay due to the great number of target DNA copies delivered into the surrogate plant.

**Table 1:** CRISPR constructs used in this work.

CRISPR Construct	Target Gene(s)	gRNAs
C1	*CYP79D1*	1A, 1B
C2	*CYP79D2*	2A, 2B
C3	*CYP79D1*, *CYP79D2*	1A, 1B, 2A, 2B

We obtained four classes of CRISPR-induced mutations for each of the target loci: bi-allelic (carrying two different mutations, one for each copy of the targeted gene); homozygous (having two identical mutations of their alleles); heterozygous (carrying one mutagenized allele and one wildtype allele); and complex (carrying more than two sequence patterns, indicating genetic mosaicism or chimerism; Frank and Chitwood, 2016). For further analysis we selected mutant lines showing bi-allelic or homozygous frameshift mutations leading to premature stop codons (**Supplementary Data File 2**), and confirmed their genotypes using Illumina amplicon sequencing (Methods). This amplicon sequencing revealed one putative dual knockout mutant as complex, bearing some wildtype alleles as well (**Supplementary Figure 5**; **Supplementary Note 2**). This result highlights the importance of thorough sequence analysis. This line was excluded from further analysis. We found no evidence of off-target mutagenesis in 60444-derived edited lines based on the sequencing of candidate off-target sites for our gRNAs (**Supplementary Tables 1**, **2**; Methods). Furthermore, cDNA of *CYP79D1* and *CYP79D2* transcripts in 60444-derived lines confirmed the expected sequences.

**Figure 2:**
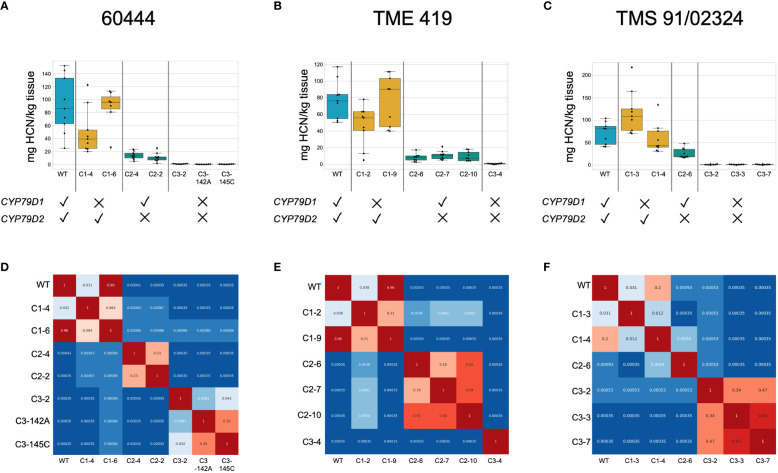
Dual knockout of *CYP79D1* and *CYP79D2* eliminates cyanide production in accessions 60444, TME 419, and TMS 91/02324. Assays were conducted 6.5, 8, and 7.5 months, respectively, after plants were transferred to soil. **(A–C)** Box and whisker plots of cyanide values in storage roots in mg HCN per kg tissue, as detected by picrate assay. Black dots are biological replicates. The median, and lower (25th percentile) and upper (75th percentile) quartiles are indicated. Whiskers define the minimum and maximum regions of the data; data points outside of these are outliers. **(D–F)** Wilcoxon Rank Sum p-values calculated from pairwise comparisons between lines of root cyanide content. Values less than 0.05, indicating the distributions of the values are statistically different between the two lines, are colored in shades of blue. Values greater than 0.05 are colored in shades of red. WT, wildtype. Edited lines are identified by the CRISPR construct with which they were modified (C1, C2, C3), followed by an index number (e.g., 142A).

To test the impact of *CYP79D* edits on cyanogen levels, we measured linamarin and lotaustralin in leaves of edited 60444 and TME 419 *in vitro* plantlets using liquid chromatography-mass spectrometry (LC-MS). Linamarin was not detected in dual knockout lines (**Supplementary Note 2**; **Supplementary Figures 6**, **7**). We also measured cyanide levels in leaves and tuberous roots of adult wildtype and mutant 60444, TME 419, and TMS 91/02324 plants, using a picrate assay (Bradbury et al., 1999). Assays were performed on greenhouse grown synchronous cohorts of plants 6–11 months after transfer to soil. Edited plants were morphologically indistinguishable from wildtypes (**Supplementary Figure 8**). Up to nine root samples from at least three plants per line were analyzed. As observed in *in vitro* plantlets, dual knockout lines showed no cyanogenic potential, and *CYP79D2* knockouts showed a more drastic reduction in cyanogenic potential relative to wildtype than did *CYP79D1* knockouts (**Figure 2**; **Supplementary Figures 9**, **10**). As cyanogens have been implicated in nitrogen storage and transport in cassava, we tested the ability of our acyanogenic plants to grow in nitrogen limited media. In this context, dual knockout plantlets displayed a morphology typical of, and indistinguishable from, wildtypes (**Supplementary Figure 11**; **Supplementary Table 3**).

We found significant differences in cyanide content between edited and unedited lines despite the well-known variability of cyanide levels between roots of the same plant and plants of the same cultivar (Cooke et al., 1978). To account for observed variability we conducted pairwise comparisons of cyanide levels using Wilcoxon Rank Sum tests (**Figure 2**; **Supplementary Figure 9**). In dual knockout lines generated from the three accessions, the zero (or very near zero) assayed cyanide levels were distinct from those of wildtype and single knockout lines. In each of the three accessions, one *CYP79D1* knockout line had cyanide levels distinct from wildtype and the other did not. This is consistent with our observation that knocking out *CYP79D1* alone does not reliably reduce cyanogenic potential below wildtype levels. All *CYP79D2* knockout lines had cyanide levels distinct from corresponding wildtype, *CYP79D1* knockouts, and dual knockouts. This is consistent with our assessment that knocking out *CYP79D2* alone provides a near complete, but not total, reduction in cyanogenic potential.

**Figure 3:**
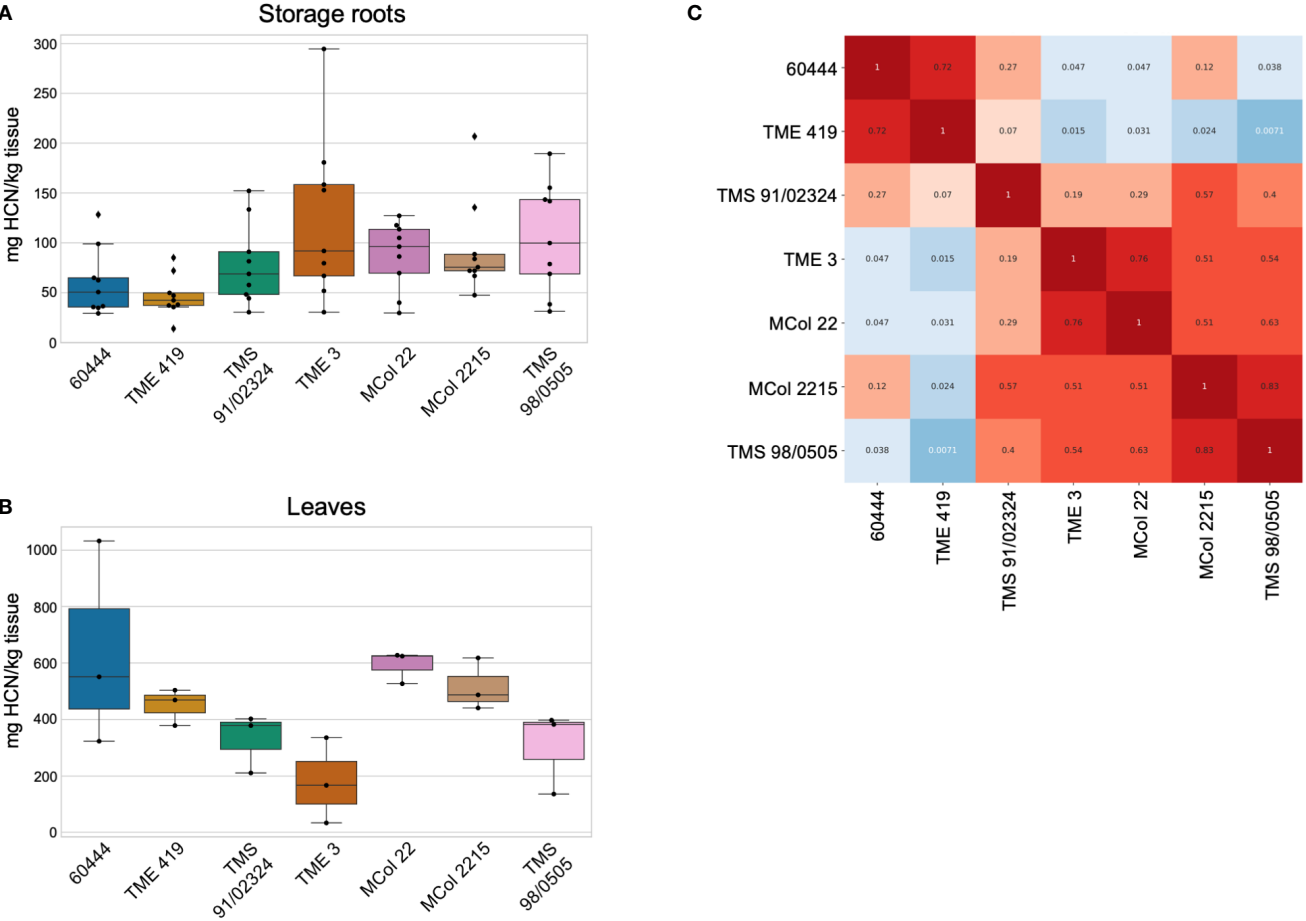
Cyanide levels from seven transformable cassava accessions. Assays were conducted seven months after plants were transferred to soil. **(A, B)** Cyanide levels in **(A)** storage roots and **(B)** leaves of the indicated cassava accessions in mg HCN per kg tissue, as detected by picrate assay. Black dots are assayed values. The median, upper, and lower quartiles are indicated. Whiskers define the minimum and maximum regions, and dots outside of these are outliers. **(A)** Root measurements. For each accession, samples were taken from a total of nine tuberous roots, from four to six plants. **(B)** Cyanide measured from leaf samples. For each accession, a total of three leaf samples were taken, from three plants. **(C)** Wilcoxon Rank Sum p-values calculated from pairwise comparisons between accessions of root cyanide content. Values less than 0.05, indicating the distributions of the values are statistically different between the two lines, are colored in shades of blue. Values greater than 0.05 are colored in shades of red.

To identify cultivars that could be prospective targets for *CYP79D* gene modification, we measured leaf and storage root cyanide content in accessions that have established transformation protocols: 60444, TME 419, TMS 91/02324, TMS 98/0505, TME 3, MCol 22, and MCol 2215 (**Figure 3**) (Li et al., 1996; Siritunga and Sayre, 2003; Taylor et al., 2012; Zainuddin et al., 2012; Chauhan et al., 2018). Genotype and environment both impact cassava root cyanide levels (Ogbonna et al., 2021). In our controlled environment, the ranges of root cyanide values largely overlapped; 60444 and TME 419 were significantly lower, but all others were not distinguishable from each other. Relative cyanide content in leaves was not predictive of relative cyanide content in roots across accessions. This result is consistent with previous work that showed weak correlation between leaf and storage root cyanide content, using a different cyanide assay method and field-grown landraces (Ospina et al., 2021).

## 3 Discussion

This study marks the first report of engineering acyanogenic plants *via* the CRISPR-Cas system. Genome editing is a powerful and heritable method to disable genes of interest for functional assessment and crop improvement. Here, we targeted cyanogenesis genes *CYP79D1* and *CYP79D2* to achieve reduction in cassava’s cyanogenic potential. We demonstrated the elimination of cyanogenic potential in eight dual knockout cassava lines. We discovered differing contributions to cyanogenesis by the two *CYP79D* genes, and observed that loss of *CYP79D2* alone is sufficient for dramatic and stable reduction in cyanide levels. These results were consistent across three different cassava accessions, in *in vitro* plantlets as well as adult plant leaves and storage roots, and *via* measurement of cyanogenic glucosides or evolved cyanide, respectively. We also assayed cyanide levels among a group of cassava lines that have elucidated transformation protocols, and are thus poised for genome engineering. The observed incongruity between relative root and leaf cyanide content indicates that these lines differ in terms of cyanogen biosynthesis, transport and/or metabolism. Understanding the mechanism and regulation of these differences may be useful for future modulation of this pathway.

The paralogous *CYP79D* genes were duplicated during the ancient paleotetraploidy in the cassava lineage (Bredeson et al., 2016). Gene duplication provides an evolutionary substrate for functional diversification (Otto and Yong, 2002), since initially redundant genes can then accumulate novel and/or complementary mutations, including variation in substrate specificity and gene expression, and may also alter the regulation of biochemical networks. Our data indicate that *CYP79D2* is likely responsible for a greater proportion of CYP79D enzymatic function than is *CYP79D1*. Differences in gene expression and/or protein sequence may explain this disparity: *CYP79D2* shows higher transcriptional activity than *CYP79D1* (Wilson et al., 2017) (**Supplementary Figure 12**), and lies in the cyanide biosynthetic gene cluster, whereas *CYP79D1* does not (Takos et al., 2011; Bredeson et al., 2021; Ogbonna et al., 2021). The 1000-bp regions upstream of the *CYP79D1* and *CYP79D2* transcript sequences have 50.17% identity; the amino acid sequences of CYP79D1 and CYP79D2 have 85.74% identity (Bredeson et al., 2021), with multiple mismatches in the transmembrane and P450 domains. There may be differences in spatial and/or temporal expression between these genes. It is also possible that genetic and metabolic feedback loops are influencing cyanogenic output of the intact *CYP79D* gene in single knockout lines. Thus, regulation and expression of the *CYP79D* and associated genes merit further study. *CYP79D2* knockouts showed a three- to ten-fold reduction of cyanide content, when comparing the mean values of these edited lines to mean values from the corresponding wildtypes. Hence, knockout of *CYP79D2* alone provides a straightforward mechanism for generating stably low-cyanide plants, if so desired.

A recent article reported a significant reduction of cyanide in the leaves of cassava plants *via* CRISPR-Cas9 targeting of the *CYP79D1* gene alone (Juma et al., 2022). The target sequence used, however, also matches a site in the *CYP79D2* gene (Bredeson et al., 2021). Thus, perhaps the degree of cyanide reduction observed in the edited lines resulted from mutations in *CYP79D2*, in addition to the reported mutations in *CYP79D1*.

Field testing of our edited lines with their corresponding wildtypes will allow well-controlled interrogation of the roles of cyanogens in cassava, including herbivory defense, stress response, nitrogen metabolism, and postharvest physiological deterioration. In addition to the *CYP79D* genes, the CRISPR-Cas9 system can be applied to modulate cyanogenic potential through modification of other genes of interest. A recent genome-wide association study, for example, identified two proteins that regulate cassava cyanide levels in storage roots (Ogbonna et al., 2021).

For all of our mutant lines, both copies of the target gene(s) are mutated, and we demonstrated that these changes to the genomic DNA are stably inherited *via* clonal propagation, which is typically used in cassava cultivation. We thus anticipate that the mutant alleles would be stably inherited *via* sexual propagation, and if crossed with other cassava, would segregate in a typical Mendelian fashion. The potential for changing a particular trait in outbred cassava varieties, without disrupting the complement of other traits for which they are preferred, is an alluring aspect of this precision breeding method, and can play a role in the maintenance of genetic diversity across the global population of cassava cultivars. For example, the editing approach demonstrated here can be applied to cassava varieties popular in regions of Africa projected to experience increased drought as a result of climate change, and hence higher cyanide risk (Nhassico et al., 2016).

With the increasing severity and frequency of drought, the ability to modulate cyanide levels in preferred cassava cultivars will increase in importance. TME 419, popular in Nigeria, is known as a low cyanide “boil and eat” cultivar. Under environmental conditions that would increase cyanogenesis, farmers and consumers using acyanogenic or reduced cyanide TME 419 would not have to alter farming and preparation practices intended for low cyanide roots. Previous work indicates that the disruption of cyanogenesis can impede cassava growth in the absence of sufficient reduced nitrogen (Siritunga and Sayre, 2004; Jørgensen et al., 2005). We observed no impact on morphology on plants in soil in glasshouse, potentially due to the provision of fertilizer containing ammoniacal nitrogen (**Supplementary Figure 8**, Methods). We also did not observe any differences between wildtype and acyanogenic plantlets grown on media with limited nitrogen (**Supplementary Figure 11**). If the complete absence of cyanogens proves deleterious to the crop in nitrogen-limited soils in the field, releasing low-cyanide *CYP79D2* knockout versions of farmer preferred cultivars may serve as a suitable approach for the reduction of cyanide risk.

Reduction of cyanogen content in cassava has the potential for broad socioeconomic benefits for cassava producers and consumers as well as positive effects on the environment. As detoxification of cassava can take days (Tewe, 1992), acyanogenic cassava can reduce processing time and labor. Women and girls, who disproportionately bear the burden of this labor, may then be at greater liberty to pursue other forms of work and education. If reduction of cyanide levels delays postharvest physiological deterioration of storage roots, the increased shelf life could economically benefit farmers and other stakeholders in the value chain (Zainuddin et al., 2018). At the industrial scale, processing of acyanogenic cassava would not release cyanide into wastewater, thereby reducing the labor and cost of wastewater treatment and/or the toxicity to local terrestrial and aquatic life (Adewoye et al., 2005; Silva et al., 2017). Moreover, acyanogenic cassava cultivars would be a boon for food safety and consumer wellness. As described above, excessive consumption of cyanide with a protein-poor diet can lead to neurological harm including decline in motor proficiency and cognitive performance, and, in severe cases, paralysis (Kashala-Abotnes et al., 2019). Acyanogenic cassava could preclude these debilitating conditions and open at-risk consumers and their would-be caretakers to other pursuits.

## 4 Materials and methods

### 4.1 gRNA and CRISPR construct design

Candidate target sequences were identified in *CYP79D1* and *CYP79D2* genes (Manes.13G094200 and Manes.12G133500, respectively, in cassava AM560-2 reference assembly v8.1, https://phytozome-next.jgi.doe.gov/info/Mesculenta_v8_1) of cassava using the online CRISPR-P 2.0 software (Liu et al., 2017). The software CasOT was used with default settings to search for potential off-targets in a 60444 genome assembly (Xiao et al., 2014; Gomez et al., 2019). Candidate gRNAs with minimal off-target potential and targeting sites approximately 500 bp apart were selected for assembly. Matching CRISPR targets in 60444 were verified by PCR amplification of targeted regions from genomic DNA extracts and Sanger sequencing (**Supplementary Table 4**).

The CRISPR-Cas9 expression entry plasmid (Thomazella et al., 2021) was re-engineered to carry the optimized gRNA scaffold with stem loop extension and A-U flip for improved Cas9 binding and gRNA transcription, respectively (Chen et al., 2014). The *BsaI* site in the backbone of the binary destination vector pCAMBIA2300 was removed *via* the QuikChange Site-Directed Mutagenesis Kit (Agilent) (Hajdukiewicz et al., 1994). The cassette carrying the CRISPR expression system was Gateway cloned into the *BsaI*-removed pCAMBIA2300 vector. The assembled binary vector with the CRISPR expression system, pCAMBIA2300 CR3-EF, requires a single cloning step for insertion of desired gRNA with white colony screen and kanamycin selection in *Escherichia coli*.

The selected CRISPR spacers were assembled into a polycistronic tRNA-gRNA (PTG) gene for multiplex targeting (Xie et al., 2015). The protocol was modified to incorporate the aforementioned stem loop extension and A-U flip. The Golden Gate cloning method was used to BsaI digest the pCAMBIA2300 CR3-EF vector and PTG ends, and then ligate the PTG into the vector. Sequences of assembled CRISPR constructs were verified *via* Sanger sequencing.

CRISPR construct activity was verified *via in planta* gemini-vector mutagenesis assay. Briefly, targeted regions were cloned into a derivative of the pLSL.D.R gemini-vector and pEAQ-HT vector maintaining replication elements for the generation of replicons bearing the target sites (Sainsbury et al., 2009; Baltes et al., 2014). Geminivirus constructs and CRISPR constructs were separately transformed into *Agrobacterium tumefaciens* strain GV3101 cultures *via* heat shock and rifampicin, gentamicin, and kanamycin selection. Transformants were grown overnight and diluted to OD600 = 0.3 each in infiltration medium (10 mM MES pH 5.6, 10 mM MgCl_2_, 150 µM acetosyringone). After incubation at room temperature for 3 h, *A. tumefaciens* cultures bearing the CRISPR construct and corresponding geminivirus-targets construct were mixed 1:1 and infiltrated into *N. benthamiana* leaves. After five days, DNA was extracted from infiltrated leaf material *via* a modified CTAB procedure (Murray and Thompson, 1980). Frozen leaf tissue was ground by 3-mm glass beads in Minibeadbeater (Biospec Products, Inc.) and resuspended in extraction buffer (1.4 M NaCl, 100 mM Tris-HCl pH 8.0, 20 mM EDTA, 2% CTAB). Following incubation at 65°C for at least 10 min, the extract was emulsified with chloroform and centrifuged at 16,000 *g* for 5 min. DNA was precipitated from the aqueous phase with an equal volume of isopropanol and centrifuged for 10 min at 4°C. The supernatant was decanted and the DNA pellet was washed with 70% ethanol. After re-centrifugation for 2 min, ethanol was removed by pipette and air drying for 5–10 min. The DNA pellet was resuspended in 1X TE Buffer. Dissolution was advanced by incubation at 60–65°C for 5 min, or overnight incubation at room temperature. Target regions were PCR amplified and run on 1.5% agarose gel (**Supplementary Table 4**). CRISPR-mediated excision of 500 bp between CRISPR targets resulted in an amplified band that was visibly smaller on the gel. 

### 4.2 Genetic transformation of cassava

*Agrobacterium*-mediated transformation was utilized to deliver CRISPR-Cas9 genome editing tools into friable embryogenic calli (FEC) of cassava accessions 60444, TME 419, and TMS 91/02324, with subsequent plant regeneration, following the protocol described by Taylor et al. (2012) and Chauhan et al. (2015). Somatic embryos were induced from leaf explants of *in vitro* micro-propagated plants by culture on Murashige and Skoog basal medium (MS) (Murashige and Skoog, 1962) supplemented with 20 g/L sucrose (MS2) plus 50 µM picloram. Pre-cotyledon stage embryos were subcultured onto Gresshoff and Doy basal medium (GD) (Gresshoff and Doy, 1974) supplemented with 20 g/L sucrose and 50 µM picloram (GD2 50P) in order to induce production of FEC. Homogenous four-month-old FEC were selected and used as target tissue for transformation with *A. tumefaciens* strain LBA4404 (Taylor et al., 2012) or AGL1, carrying CRISPR constructs targeting *CYP79D1* (C1), *CYP79D2* (C2), and both genes (C3). In some cases, infection was performed using sonication with a Branson 3510-DTH Ultrasonic Cleaner for three seconds. Transgenic tissues were selected and proliferated on GD2 50P containing paromomycin, prior to regeneration of embryos on MS2 medium supplemented with naphthalene acetic acid (NAA). Somatic embryos were germinated on MS2 medium containing 6-benzylaminopurine (BAP). Regenerated plants were maintained on MS2 in Phytatrays II (Sigma-Aldrich, St. Louis, MO), incubated at 28°C in high light (90–150 μmol m^-2^ s^-1^ for 16 h light/8 h dark conditions) and subcultured every 3 weeks.

### 4.3 Plant growth and maintenance

Plantlets were maintained in MS2 agar medium for stem elongation and stable growth. Well-developed growing shoots were maintained in growth chambers in Phytatrays, one to two shoots per tray, following the conditions described by Taylor et al. (2012). Regenerated plants were micro-propagated and rooted in MS2 medium containing 2.2 g/L phytagel at two to three plantlets per petri dish. For the nitrogen limitation experiment shown in **Supplementary Figure 11**, plantlets were grown in standard MS2 medium; MS2 medium at ½ X concentration; or MS2 medium with ½ the amount of ammonium nitrate (see **Supplementary Table 3**). After three weeks rooted plantlets were synchronously transferred into soil (BM7 45% bark mix, Berger) in 3” square (0.37 L^3^) Kord pots and grown in a glasshouse. During soil transfer, pots were subirrigated with an aqueous solution containing, per gallon, Gnatrol WDG larvicide (Valent) at the label rate, ½ tsp Jack’s Professional LX 15-5-15 Ca-Mg fertilizer (JR Peters, Inc.), ¼ tsp Jack’s Professional M.O.S.T. mix of soluble traces (JR Peters, Inc.), and ½ tsp Sprint 330 (Becker Underwood). Plants were transferred to soil and watered, and the pots placed in trays with drainage holes. The trays were covered with a low (2”) dome and kept on a heating pad set to 80°F in a misting bench for 100% humidity, under 40% white shade cloth. After 8–16 days, the low domes were replaced with 6” high vented domes and the trays moved into a room without shade, with misting three times per day. The domes were removed after 9–11 days and plants placed approximately six per tray in 28-pocket spacing trays. Some plants that were lagging were kept under non-vented high domes for longer periods. For the first four weeks after transfer to soil, plants were watered with Jack’s Peat Lite 15-16-17 (JR Peters, Inc.) at 200 ppm approximately three times per week, and thereafter with Jack’s Blossom Booster 10-30-20 (JR Peters, Inc.) at 100 ppm two times per week (Taylor et al., 2012). Plants were watered with tap water on days fertilizer was not administered.

### 4.4 Sequence analysis

Putative transgenic lines (based on growth on antibiotic) were genotyped at the target loci. Genomic DNA extraction, PCR amplification, and Sanger sequencing were conducted as described in Gomez et al. (2019). PCR amplification and Sanger sequencing were performed using the gemini-CYP79D primers (**Supplementary Table 4**). The genotypes of all plants sampled in the **Figure 2** picrate assay were confirmed by DNA sequence analysis of the target loci.

Percent identity of the *CYP79D1* and *CYP79D2* promoter sequences and protein amino acid sequences was evaluated using sequences derived from the cassava v6.1 genome assembly (Bredeson et al., 2016). Sequences were aligned for analysis using Clustal Omega under default settings (Madeira et al., 2019). Protein domains were predicted using SMART (Letunic et al., 2021).

#### 4.4.1 Amplicon sequencing of selected CRISPR-Cas9 edited lines

For each line, leaf samples from two parts of an individual plant were collected for DNA extraction. PCR reactions for amplicon sequencing were performed using Phusion HF Polymerase and amplicon sequencing primers (**Supplementary Table 4**), and amplified for 25 cycles. Samples were deep sequenced on an Illumina MiSeq using 300 bp paired-end reads to a depth of at least 10,000 reads per sample. Cortado (https://github.com/staciawyman/cortado) was used to analyze editing outcomes. Briefly, reads were adapter trimmed and then merged using overlap into single reads. These joined reads were then aligned to the target sequence using NEEDLE (Li et al., 2015) to identify any insertions or deletions (indels) overlapping the targeted cut site. Genotypes found in less than 1% of reads were considered to be PCR or sequencing errors.

#### 4.4.2 Off-target analysis

Identification of potential off-target loci was performed using CasOT software and a reference-based genome assembly of accession 60444, as described previously (Xiao et al., 2014; Gomez et al., 2019). Sites that contained the protospacer adjacent motif (PAM) region of the gRNA were selected and ranked according to sequence similarity to the target site, and the 2–3 highest ranking potential off-targets (those with the fewest mismatches to the gRNA spacer) for each gRNA were selected for sequence analysis (**Supplementary Table 1**). Genomic DNA was extracted from cassava leaves using the modified CTAB protocol described above. The selected potential off-target regions were amplified using Phusion polymerase (New England Biolabs [NEB]) and touchdown PCR (TD-PCR) (Korbie and Mattick, 2008), Phusion polymerase and 30 cycles of PCR with an annealing temperature of 63°C, or OneTaq Quick-Load 2X Master Mix with Standard Buffer (NEB) with 35 PCR cycles and an annealing temperature of 47°C. PCR primer sequences are listed in **Supplementary Table 4**. The TD-PCR protocol began with an annealing temperature of Tm + 10°C for the first cycling phase (Tm calculated by NEB Tm Calculator tool). The annealing temperature was then decreased by 1°C per cycle until the primers’ Tm was reached, followed by 20 or 25 cycles using the primer Tm as the annealing temperature. PCR amplicons were visualized using gel electrophoresis, then the remaining reaction was purified for sequencing *via* the AccuPrep PCR/Gel Purification Kit (Bioneer), the Monarch PCR & DNA Cleanup Kit (NEB), or SPRI magnetic nucleic acid purification beads (UC Berkeley DNA Sequencing Facility). We sequenced purified amplicons containing potential off‐targets using Sanger sequencing. Putative off-target loci were then examined in SnapGene for potential sequence discrepancy with the 60444 reference sequence.

#### 4.4.3 RT-PCR

Cassava cDNA was generated using the Spectrum Plant Total RNA Kit (Sigma-Aldrich) following Protocol A and performing On-Column DNase Digestion. Concentrations of RNA extracts were measured by NanoDrop One (Thermo Fisher Scientific). Quality of RNA was examined by first denaturing aliquots at 70°C for 5 min (followed by 4°C on ice), then electrophoresing 200 ng of RNA on 1.5% UltraPure Agarose (Invitrogen). 450–1000 ng of RNA was added to SuperScript III Reverse Transcriptase (Invitrogen) reaction mix with Oligo(dT)_20_. Reaction was run for 60 min at 50°C followed by RNase H treatment. Primers were designed to amplify the *CYP79D* transcripts from the 5’ UTR to the 3’ UTR (**Supplementary Table 4**). 2 µL of cDNA mix was added to 50 µL Phusion High-Fidelity DNA Polymerase (NEB) reaction mix. cDNA was amplified for 35 cycles. PCR reactions were run on 1.5% agarose and desired bands were extracted. Amplicons were cloned into the Zero Blunt PCR Cloning Kit (Thermo Fisher Scientific) and 10–12 colonies subsequently sequenced *via* the UC Berkeley DNA Sequencing Facility.

### 4.5 Measurement of cyanogenic potential

#### 4.5.1 Measurement of cyanogens from *in vitro* plantlets

We used LC-MS to measure linamarin and lotaustralin in *in vitro* plantlets. Regenerated transgenic plants were micro-propagated and established in MS2 medium at two plantlets per Phytatray in a growth chamber at 28°C +/- 1°C, 41% relative humidity, 120–150 μmol/m^2^/s light for 16 h light/8 h dark conditions. After four to five weeks plants were ready for tissue sampling. One leaf was harvested from each plantlet and stored in a plastic bag on ice until extraction, approximately 1–3 h later. For biological replicates, we harvested tissue from three plantlets per line.

Approximately 20 or 30 mg of leaf tissue was excised from fresh leaves and placed in a 1.5-mL tube (Safe-Lock, Eppendorf) with 600 or 900 µL of 85% MeOH warmed to approximately 68°C. Sample weight was recorded. Negative controls contained no tissue. A cap lock was added and the tube was floated in boiling water for 3 min, then returned to ice. One to three tubes were boiled at a time. Cooled tubes were spun down briefly. A 1:10 or 1:20 dilution was prepared from each extract, pipetted up and down to mix, and spun through a 0.45-µM spin filter (Ultrafree MC HV Durapore PVDF, EMD Millipore) for 2 min, 10,000 x *g*, 4°C. 20 µL of filtered extract was placed in a glass autosampler vial with insert (Fisher Scientific), and the LC-MS run begun the same day. Three samples were submitted from each extract, for technical replicates.

To facilitate absolute quantitation, standard stocks were prepared from solid linamarin (Cayman Chemical, purity ≥98%) and lotaustralin (Millipore Sigma, purity ≥95%) resuspended in 85% MeOH to 3 or 4 mM, aliquoted into dark glass vials, and stored at –20°C. On the day of assay, these standards were further diluted in 85% MeOH and submitted for LC-MS analysis. Lotaustralin standards ranged in concentration from 0.01 to 1 µM, and linamarin from 0.05 to 5 µM. Submitted standard samples contained both linamarin and lotaustralin in known quantities. As with extracts, three technical replicates were performed for each standard. To buffer against any potential position/timing effects, samples were analyzed by LC-MS in three consecutive cohorts, where each cohort had one technical replicate from each sample.

Samples of cassava extracts were analyzed using a liquid chromatography (LC) system (1200 series, Agilent Technologies, Santa Clara, CA) that was connected in line with an LTQ-Orbitrap-XL mass spectrometer equipped with an electrospray ionization (ESI) source (Thermo Fisher Scientific, San Jose, CA). The LC system contained the following modules: G1322A solvent degasser, G1311A quaternary pump, G1316A thermostatted column compartment, and G1329A autosampler (Agilent). The LC column compartment was equipped with a reversed-phase analytical column (length: 150 mm, inner diameter: 1.0 mm, particle size: 5 µm, Viva C18, Restek, Bellefonte, PA). Acetonitrile, formic acid (Optima LC-MS grade, 99.5+%, Fisher, Pittsburgh, PA), and water purified to a resistivity of 18.2 MΩ·cm (at 25°C) using a Milli-Q Gradient ultrapure water purification system (Millipore, Billerica, MA) were used to prepare LC mobile phase solvents. Solvent A was 99.9% water/0.1% formic acid and solvent B was 99.9% acetonitrile/0.1% formic acid (volume/volume). The elution program consisted of isocratic flow at 2% B for 2 min, a linear gradient to 6% B over 1 min, a linear gradient to 90% B over 0.5 min, isocratic flow at 90% B for 4.5 min, a linear gradient to 2% B over 0.5 min, and isocratic flow at 2% B for 16.5 min, at a flow rate of 200 µL/min. The column compartment was maintained at 40°C and the sample injection volume was 10 µL. Full-scan mass spectra were acquired in the positive ion mode over the range of mass-to-charge ratio (*m*/*z*) = 200 to 800 using the Orbitrap mass analyzer, in profile format, with a mass resolution setting of 100,000 (at *m*/*z* = 400, measured at full width at half-maximum peak height, FWHM). For tandem mass spectrometry (MS/MS or MS^2^) analysis, selected precursor ions were fragmented using collision-induced dissociation (CID) under the following conditions: MS/MS spectra acquired using the linear ion trap, in centroid format, normalized collision energy: 35%, activation time: 30 ms, and activation Q: 0.25. Mass spectrometry data acquisition and analysis were performed using Xcalibur software (version 2.0.7, Thermo Fisher Scientific).

To convert LC-MS concentration values in µM to grams per kg fresh weight (fw), the following formula was used:


μM×dilution factor×extraction vol (L)×molecular weight (g/mol)/mg fw=g/kg fresh weight

where the molecular weight of linamarin is 247.248 g/mol and of lotaustralin is 261.272 g/mol. Concentration values reported as <LLOQ (below the lower limit of quantification) were treated as 0 µM.

#### 4.5.2 Measurement of cyanide from adult cassava plants

Each assay cohort was synchronously grown from *in vitro* plantlets transferred to soil on the same day. Plants were collected from the glasshouse and assayed on the same day, six to 11 months after transfer to soil. The height of each plant’s stem(s) was measured from the topsoil to the apical meristem in a direct line, without forcing the stems to bend, to the nearest 0.5 cm. Leaf and tuberous root samples were collected for cyanide content analysis *via* the picrate paper assay using Konzo Kit A from Australia National University Konzo Prevention Group (Bradbury, Egan, and Bradbury 1999) (https://biology.anu.edu.au/research/resources-tools/konzo-kits). Leaf samples were collected from three plants of each line. The third, fourth, and fifth expanded leaves from the top were cut perpendicular to the midribs into 0.5 cm wide pieces with clean scissors. Leaf cuts were immediately ground with a mortar and pestle. One hundred milligrams of ground leaf was loaded into a vial containing buffer paper, and 1 mL of water and the cyanide indicator paper were added immediately and the vial capped. For negative controls, no tissue was added to a vial. Positive controls were conducted using the standard provided with the kit. Sample vials were incubated overnight (minimum 12 h) at room temperature. Up to nine tuberous roots were collected from each line, with no more than three roots coming from a single plant. To buffer against any cyanide variation over the course of processing samples, root collection was staggered among groups to three to five roots per line at a time. Roots of minimum 1 cm in diameter were collected, washed, and photographed by a ruler for scale. Each root was cut at its widest section, and a 1.5 mm slice was taken crosswise *via* a kitchen mandoline. The peel (rind) was removed and 100 mg of tuberous root loaded into a vial and sealed as described above.

Indicator papers were removed from vials and compared to a cyanide color chart for an approximate cyanide content reference. These papers were then placed in 15 mL culture tubes and completely immersed in 5 mL of water. Solutions were incubated at room temperature for 30–60 min with occasional gentle stirring. The absorbance of each pipette-mixed solution was measured at 510 nm using an Ultrospec 3000 UV/Visible Spectrophotometer (Pharmacia Biotech). Absorbance was normalized to the value of the negative control (no plant sample). Absorbance was multiplied by 396 to acquire the total cyanide content in ppm (equivalent to mg HCN per kg of tissue).

#### 4.5.3 Statistical analyses

Box and whisker plots were generated for cyanide measurements from picrate assays. The box of each plot represents the interquartile range (IQR) which is bounded by a lower quartile (Q1, 25th percentile) and an upper quartile (Q3, 75th percentile) of the data. The whiskers of each plot are defined as the approximate minima and maxima of the data, with the minimum data value defined as Q1 – 1.5 × IQR, and the maximum defined as Q3 + 1.5 × IQR. Data outside of the maximum and minimum values were considered outliers.

The Wilcoxon rank-sum statistic (also known as the Mann–Whitney *U* test) was performed on the picrate data to test whether there were statistically significant differences between the various lines, using pairwise comparisons. This method was used primarily due to the nonparametric and continuous nature of our picrate data.

For data shown in **Figures 2**, **3** and **Supplementary Figure 9**, two-group Wilcoxon rank-sum statistical comparisons between all lines were performed *via* SciPy’s stats.ranksum function (https://docs.scipy.org/doc/scipy/reference/generated/scipy.stats.ranksums.html; Scipy v1.4.1), which uses a normal approximation of the rank sum. The p-values of each two-group test were compiled to form heatmaps. In these heatmaps, p-values less than 0.05 were considered significant and shaded blue, indicating that for two compared groups, the data appeared to come from two separate distributions. P-values greater than 0.05 were shaded red and indicated that for two compared groups, the data appeared as if drawn from the same distribution, demonstrating statistical insignificance from each other. Software versions used in these statistical analyses: Python v3.7.13; Numpy v1.21.6; Pandas v1.3.5; Matplotlib v3.2.2. Plots in **Figures 2**, **3** and **Supplementary Figure 9** were generated in Google Colab notebooks with the Seaborn data visualization library (v0.11.2).

For data shown in **Supplementary Figure 10**, Wilcoxon rank-sum statistical comparisons were calculated as described above using R studio v1.1.456. Figures were generated using the ggplot2 package in R studio (Wickham, 2016).

## Data availability statement

The data produced in this study are available in the article and **Supplementary Material**. The raw Illumina sequence data generated for this study were deposited in the NCBI Sequence Read Archive (SRA), under BioProject PRJNA906627. Vectors are available upon request to BS stask@berkeley.edu. Plants are available upon request to M-JC mjcho1223@berkeley.edu.

## Author contributions

Designed the project: MG, NT, BS, M-JC, DR, JL. Molecular biology lead: MG. Gemini-vector transient assay: MG, AS. Cassava transformation: BG, RC. Genotyping of on- and off-target sites: MG, KB, SL, JM. Extractions for LC-MS: MG, BG, JL. LC-MS: AI. LC-MS plots: JL. Picrate assays: MG, KB, SL, NK, JL. Picrate assay plots and statistical analysis: KB, NK. Illumina amplicon data analysis: SW. Project leadership: JL, with co-PIs BS, M-JC, DR; Danforth Center lead NT. Wrote the paper: MG and JL; with contributions by KB, BG, AI, SL, SW, NT, M-JC; and edited by NT, M-JC, and DR. All authors contributed to the article and approved the submitted version.

## Supplementary material

A **Supplementary Materials** file including Supplementary Notes 1 and 2, Supplementary Figures 1–12, Supplementary Tables 1–4, and legends for Supplementary Data Files 1–4.

Supplementary Data File 1 Genotypes of CRISPR-Cas9-edited 60444, TME 419, and TMS 91/02324 lines.

Supplementary Data File 2 Predicted CYP79D amino acid sequences from selected 60444, TME 419, and TMS 91/02324 lines.

Supplementary Data File 3 LC-MS, picrate, and height readings.

Supplementary Data File 4 Python notebook for Figures 2, 3 and Supplementary Figure 9.
