# Supplementary material for: CRISPR-Cas9-mediated knockout of CYP79D1 and CYP79D2 in cassava attenuates toxic cyanogen production
Source: Front Plant Sci. 2023 Mar 17;13:1079254. doi: 10.3389/fpls.2022.1079254 (PMC10064795; doi:10.3389/fpls.2022.1079254)
Supplement: Supplementary file 1 — A Supplementary Materials file including Supplementary Notes 1 and 2, Supplementary Figures 1–12, Supplementary Tables 1–4, and legends for Supplementary Data Files 1–4. [file DataSheet_1.zip › Gomezetal_Supplement_all_final/SupplementaryDataFile2_Gomezetal.pdf]

>60444_CYP79D1_WT
MAMNVSTTIGLLNATSFASSSSINTVKILFVTLFISIVSTIVKLQKSAANKEGSKKLPLPPGPTPWPLIG
NIPEMIRYRPTFRWIHQLMKDMNTDICLIRFGRTNFVPISCPVLAREILKKNDAIFSNRPKTLSAKSMSG
GYLTTIVVPYNDQWKKMRKILTSEIISPARHKWLHDKRAEEADNLVFYIHNQFKANKNVNLRTATRHYGG
NVIRKMVFSKRYFGKGMPDGGPGPEEIEHIDAVFTALKYLYGFCISDFLPFLLGLDLDGQEKFVLDANKT
IRDYQNPLIDERIQQWKSGERKEMEDLLDVFITLKDSDGNPLLTPDEIKNQIAEIMIATVDNPSNAIEWA
MGEMLNQPEILKKATEELDRVVGKDRLVQESDIPNLDYVKACAREAFRLHPVAHFNVPHVAMEDTVIGDY
FIPKGSWAVLSRYGLGRNPKTWSDPLKYDPERHMNEGEVVLTEHELRFVTFSTGRRGCVASLLGSCMTTM
LLARMLQCFTWTPPANVSKIDLAETLDELTPATPISAFAKPRLAPHLYPTSP*
>60444_CYP79D1_C1-4
MAMNVSTTIGLLNATSFASSSSINTVKILFVTLFISIVSTIVKLQKSAANKGR*
>60444_CYP79D1_C1-6
MAMNVSTTIGLLNATSFASSSSINTVKILFVTLFISIVSTIVKLQKSAANKGR*
>60444_CYP79D1_C3-2_1
MAMNVSTTIGLLNATSFASSSSINTVKILFVTLFISIVSTIVKLQKSAANNGR*
>60444_CYP79D1_C3-2_2
MAMNVSTTIGLLNATSFASSSSINTVKILFVTLFISIVSTIVKLQKSAANKGR*
>60444_CYP79D1_C3-142A_1
MAMNVSTTIGLLNATSFASSSSINTVKILFVTLFISIVSTIVKLQKSAARKVARNSHSLLALLHGHSSET
SRK*
>60444_CYP79D1_C3-142A_2
MAMNVSTTIGLLNATSFASSSSINTVKILFVTLFISIVSTIVKLQKSAANKGR*
>60444_CYP79D1_C3-145C
MAMNVSTTIGLLNATSFASSSSINTVKILFVTLFISIVSTIVKLQKSAANKGR*
>60444_CYP79D2_WT
MAMNVSTTATTTASFASTSSMNNTAKILLITLFISIVSTVIKLQKRASYKKASKNFPLPPGPTPWPLIGN
IPEMIRYRPTFRWIHQLMKDMNTDICLIRFGKTNVVPISCPVIAREILKKHDAVFSNRPKILCAKTMSGG
YLTTIVVPYNDQWKKMRKVLTSEIISPARHKWLHDKRAEEADQLVFYINNQYKSNKNVNVRIAARHYGGN
VIRKMMFSKRYFGKGMPDGGPGPEEIMHVDAIFTALKYLYGFCISDYLPFLEGLDLDGQEKIVLNANKTI
RDLQNPLIEERIQQWRSGERKEMEDLLDVFITLQDSDGKPLLNPDEIKNQIAEIMIATIDNPANAVEWAM
GELINQPELLAKATEELDRVVGKDRLVQESDIPNLNYVKACAREAFRLHPVAYFNVPHVAMEDAVIGDYF
IPKGSWAILSRYGLGRNPKTWPDPLKYDPERHLNEGEVVLTEHDLRFVTFSTGRRGCVAALLGTTMITMM
LARMLQCFTWTPPPNVTRIDLSENIDELTPATPITGFAKPRLAPHLYPTSP*
>60444_CYP79D2_C2-2_1
MAMNVSTTATTTASFASTSSMNNTAKILLITLFISIVSTVIKLQKRASYKKASKNFPLPPPWPLIGNIPE
MIRYRPTFRWIHQLMKDMNTDICLIRFGKTNVVPISCPVIAREILKKHDAVFSNRPKILCAKTMSGGYLT
TIVVPYNDQWKKMRKVLTSEIISPARHKWLHDKRAEEADQLVFYINNQYKSNKNVNVSLRQGITVEM*
>60444_CYP79D2_C2-2_2
MAMNVSTTATTTASFASTSSMNNTAKILLITLFISIVSTVIKLQKRASYKKASKNFPLPPSMATHRKHP*
>60444_CYP79D2_C2-4
MAMNVSTTATTTASFASTSSMNNTAKILLITLFISIVSTVIKLQKRASYKKASKNFPLPPGSDSMATHRK
HP*
>60444_CYP79D2_C3-2_1
MAMNVSTTATTTASFASTSSMNNTAKILLITLFISIVSTVIKLQKRASYKKASKNFPLPPGPTPWPLIGN
IPEMIRYRPTFRWIHQLMKDMNTDICLIRFGKTNVVPISCPVIAREILKKHDAVFSNRPKILCAKTMSGG
YLTTIVVPYNDQWKKMRKVLTSEIISPARHKWLHDKRAEEADQLVFYINNQYKSNKNVNVRNHALRWKCD
QKDDV*
>60444_CYP79D2_C3-2_2
MAMNVSTTATTTASFASTSSMNNTAKILLITLFISIVSTVIKLQKRASYKKASKNFPLPPGPTPWPLIGN
IPEMIRYRPTFRWIHQLMKDMNTDICLIRFGKTNVVPISCPVIAREILKKHDAVFSNRPKILCAKTMSGG

YLTTIVVPYNDQWKKMRKVLTSEIISPARHKWLHDKRAEEADQLVFYINNQYKSNKNVNVRICGKALRWK
CDQKDDV*
>60444_CYP79D2_C3-142A
MAMNVSTTATTTASFASTSSMNNTAKILLITLFISIVSTVIKLQKRASYKKASKNFPLPPGPTPWPLIGN
IPEMIRYRPTFRWIHQLMKDMNTDICLIRFGKTNVVPISCPVIAREILKKHDAVFSNRPKILCAKTMSGG
YLTTIVVPYNDQWKKMRKVLTSEIISPARHKWLHDKRAEEADQLVFYINNQYKSNKNVNVRICGKALRWK
CDQKDDV*
>60444_CYP79D2_C3-145C
MAMNVSTTATTTASFASTSSMNNTAKILLITLFISIVSTVIKLQKRASYKKASKNFPLPPGPTPWPLIGN
IPEMIRYRPTFRWIHQLMKDMNTDICLIRFGKTNVVPISCPVIAREILKKHDAVFSNRPKILCAKTMSGG
YLTTIVVPYNDQWKKMRKVLTSEIISPARHKWLHDKRAEEADQLVFYINNQYKSNKNVNVRICGKALRWK
CDQKDDV*
>TME419_CYP79D1_WT
MAMNVSTTIGLLNATSFASSSSINTVKILFVTLFISIVSTIVKLQKSAANKEGSKKLPLPPGPTPWPLIG
NIPEMIRYRPTFRWIHQLMKDMNTDICLIRFGRTNFVPISCPVLAREILKKNDAIFSNRPKTLSAKSMSG
GYLTTIVVPYNDQWKKMRKILTSEIISPARHKWLHDKRAEEADNLVFYIHNQFKANKNVNLRTATRHYGG
NVIRKMVFSKRYFGKGMPDGGPGPEEIEHIDAVFTALKYLYGFCISDFLPFLLGLDLDGQEKFVLDANKT
IRDYQNPLIDERIQQWKSGERKEMEDLLDVFITLKDSDGNPLLTPDEIKNQIAEIMIATVDNPSNAIEWA
MGEMLNQPEILKKATEELDRVVGKDRLVQESDIPNLDYVKACAREAFRLHPVAHFNVPHVAMEDTVIGDY
FIPKGSWAVLSRYGLGRNPKTWSDPLKYDPERHMNEGEVVLTEHELRFVTFSTGRRGCVASLLGSCMTTM
LLARMLQCFTWTPPANVSKIDLAETLDELTPATPISAFAKPRLAPHLYPTSP*
>TME419_CYP79D1_C1-2
MAMNVSTTIGLLNATSFASSSSINTVKILFVTLFISIVSTIVKLQKSAANNGR*
>TME419_CYP79D1_C1-9
MAMNVSTTIGLLNATSFASSSSINTVKILFVTLFISIVSTIVKLQKSAANKGR*
>TME419_CYP79D1_C1-10_1
MAMNVSTTIGLLNATSFASSSSINTVKILFVTLFISIVSTIVKLQKSAANKGR*
>TME419_CYP79D1_C1-10_2
MAMNVSTTIGLLNATSFASSSSINTVKILFVTLFISIVSTIVKLQKSAAKGR*
>TME419_CYP79D1_C3-4_1
MAMNVSTTIGLLNATSFASSSSINTVKILFVTLFISIVSTIVKLQKSAANALRRECDQKNGVQQEILRQG
NAGRRTRA*
>TME419_CYP79D1_C3-4_2
MAMNVSTTIGLLNATSFASSSSINTVKILFVTLFISIVSTIVKLQKSAANKVARNSHSLLALLHGHSSET
SRK*
>TME419_CYP79D1_C3-44
MAMNVSTTIGLLNATSFASSSSINTVKILFVTLFISIVSTIVKLQKSAAR*
>TME419_CYP79D2_WT
MAMNVSTTATTTASFASTSSMNNTAKILLITLFISIVSTVIKLQKRASYKKASKNFPLPPGPTPWPLIGN
IPEMIRYRPTFRWIHQLMKDMNTDICLIRFGKTNVVPISCPVIAREILKKHDAVFSNRPKILCAKTMSGG
YLTTIVVPYNDQWKKMRKVLTSEIISPARHKWLHDKRAEEADQLVFYINNQYKSNKNVNVRIAARHYGGN
VIRKMMFSKRYFGKGMPDGGPGPEEIMHVDAIFTALKYLYGFCISDYLPFLEGLDLDGQEKIVLNANKTI
RDLQNPLIEERIQQWRSGERKEMEDLLDVFITLQDSDGKPLLNPDEIKNQIAEIMIATIDNPANAVEWAM
GELINQPELLAKATEELDRVVGKDRLVQESDIPNLNYVKACAREAFRLHPVAYFNVPHVAMEDAVIGDYF
IPKGSWAILSRYGLGRNPKTWPDPLKYDPERHLNEGEVVLTEHDLRFVTFSTGRRGCVAALLGTTMITMM
LARMLQCFTWTPPPNVTRIDLSENIDELTPATPITGFAKPRLAPHLYPTSP*
>TME419_CYP79D2_C2-6_1
MAMNVSTTATTTASFASTSSMNNTAKILLITLFISIVSTVIKLQKRASYKKASKNFPLPPRSDSMATHRK
HP*
>TME419_CYP79D2_C2-6_2

MAMNVSTTATTTASFASTSSMNNTAKILLITLFISIVSTVIKLQKRASYKKASKNFPLPSDSMATHRKHP
*
>TME419_CYP79D2_C2-7_1
MAMNVSTTATTTASFASTSSMNNTAKILLITLFISIVSTVIKLQKRASYKKASKNFPLPPRLHGHSSETS
LK*
>TME419_CYP79D2_C2-7_2
MAMNVSTTATTTASFASTSSMNNTAKILLITLFISIVSTVIKLQKRASYKKASKNFPLPPLHGHSSETSL
K*
>TME419_CYP79D2_C2-10_1
MAMNVSTTATTTASFASTSSMNNTAKILLITLFISIVSTVIKLQKRASYKKASKNFPLPPRSDSMATHRK
HP*
>TME419_CYP79D2_C2-10_2
MAMNVSTTATTTASFASTSSMNNTAKILLITLFISIVSTVIKLQKRASYKKASKNFPLPPVRLHGHSSET
SLK*
>TME419_CYP79D2_C3-4_1
MAMNVSTTATTTASFASTSSMNNTAKILLITLFISIVSTVIKLQKRASYKKASKNFPLATHRKHP*
>TME419_CYP79D2_C3-4_2
MAMNVSTTATTTASFASTSSMNNTAKILLITLFISIVSTVIKLQKRASYKKASKNFPLPPGPTPWPLIGN
IPEMIRYRPTFRWIHQLMKDMNTDICLIRFGKTNVVPISCPVIAREILKKHDAVFSNRPKILCAKTMSGG
YLTTIVVPYNDQWKKMRKVLTSEIISPARHKWLHDKRAEEADQLVFYINNQYKSNKNVNVRICGKALRWK
CDQKDDV*
>TME419_CYP79D2_C3-44_1
MAMNVSTTATTTASFASTSSMNNTAKILLITLFISIVSTVIKLQKRASYKKASKNFPLPPVRLHGHSSET
SLK*
>TME419_CYP79D2_C3-44_2
MAMNVSTTATTTASFASTSSMNNTAKILLITLFISIVSTVIKLQKRASYKKASKNFPLIGNIPEMIRYRP
TFRWIHQLMKDMNTDICLIRFGKTNVVPISCPVIAREILKKHDAVFSNRPKILCAKTMSGGYLTTIVVPY
NDQWKKMRKVLTSEIISPARHKWLHDKRAEEADQLVFYINNQYKSNKNVNVRQGITVEM*
>TMS91/02324_CYP79D1_WT
MAMNVSTTIGLLNATSFASSSSINTVKILFVTLFISIVSTIVKLQKSAANKEGSKKLPLPPGPTPWPLIG
NIPEMIRYRPTFRWIHQLMKDMNTDICLIRFGRTNFVPISCPVLAREILKKNDAIFSNRPKTLSAKSMSG
GYLTTIVVPYNDQWKKMRKILTSEIISPARHKWLHDKRAEEADNLVFYIHNQFKANKNVNLRTATRHYGG
NVIRKMVFSKRYFGKGMPDGGPGPEEIEHIDAVFTALKYLYGFCISDFLPFLLGLDLDGQEKFVLDANKT
IRDYQNPLIDERIQQWKSGERKEMEDLLDVFITLKDSDGNPLLTPDEIKNQIAEIMIATVDNPSNAIEWA
MGEMLNQPEILKKATEELDRVVGKDRLVQESDIPNLDYVKACAREAFRLHPVAHFNVPHVAMEDTVIGDY
FIPKGSWAVLSRYGLGRNPKTWSDPLKYDPERHMNEGEVVLTEHELRFVTFSTGRRGCVASLLGSCMTTM
LLARMLQCFTWTPPANVSKIDLAETLDELTPATPISAFAKPRLAPHLYPTSP*
>TMS91/02324_CYP79D1_C1-3_1
MAMNVSTTIGLLNATSFASSSSINTVKILFVTLFISIVSTIVKLQKSAANNGR*
>TMS91/02324_CYP79D1_C1-3_2
MAMNVSTTIGLLNATSFASSSSINTVKILFVTLFISIVSTIVKLQKSAANKGR*
>TMS91/02324_CYP79D1_C1-4
MAMNVSTTIGLLNATSFASSSSINTVKILFVTLFISIVSTIVKLQKSAANNGR*
>TMS91/02324_CYP79D1_C3-2_1
MAMNVSTTIGLLNATSFASSSSINTVKILFVTLFISIVSTIVKLQKSAANRKVARNSHSLLALLHGHSSE
TSRK*
>TMS91/02324_CYP79D1_C3-2_2
MAMNVSTTIGLLNATSFASSSSINTVKILFVTLFISIVSTIVKLQKSAANKGR*
>TMS91/02324_CYP79D1_C3-3
MAMNVSTTIGLLNATSFASSSSINTVKILFVTLFISIVSTIVKLQKSAANKGR*

>TMS91/02324_CYP79D1_C3-7_1
MAMNVSTTIGLLNATSFASSSSINTVKILFVTLFISIVSTIVKLQKSAAKGR*
>TMS91/02324_CYP79D1_C3-7_2
MAMNVSTTIGLLNATSFASSSSINTVKILFVTLFISIVSTIVKLQKSAARKVARNSHSLLALLHGHSSET
SRK*
>TMS91/02324_CYP79D2_WT
MAMNVSTTATTTASFASTSSMNNTAKILLITLFISIVSTVIKLQKRASYKKASKNFPLPPGPTPWPLIGN
IPEMIRYRPTFRWIHQLMKDMNTDICLIRFGKTNVVPISCPVIAREILKKHDAVFSNRPKILCAKTMSGG
YLTTIVVPYNDQWKKMRKVLTSEIISPARHKWLHDKRAEEADQLVFYINNQYKSNKNVNVRIAARHYGGN
VIRKMMFSKRYFGKGMPDGGPGPEEIMHVDAIFTALKYLYGFCISDYLPFLEGLDLDGQEKIVLNANKTI
RDLQNPLIEERIQQWRSGERKEMEDLLDVFITLQDSDGKPLLNPDEIKNQIAEIMIATIDNPANAVEWAM
GELINQPELLAKATEELDRVVGKDRLVQESDIPNLNYVKACAREAFRLHPVAYFNVPHVAMEDAVIGDYF
IPKGSWAILSRYGLGRNPKTWPDPLKYDPERHLNEGEVVLTEHDLRFVTFSTGRRGCVAALLGTTMITMM
LARMLQCFTWTPPPNVTRIDLSENIDELTPATPITGFAKPRLAPHLYPTSP*
>TMS91/02324_CYP79D2_C2-6_1
MAMNVSTTATTTASFASTSSMNNTAKILLITLFISIVSTVIKLQKRASYKKASKNFPRPTPWPLIGNIPE
MIRYRPTFRWIHQLMKDMNTDICLIRFGKTNVVPISCPVIAREILKKHDAVFSNRPKILCAKTMSGGYLT
TIVVPYNDQWKKMRKVLTSEIISPARHKWLHDKRAEEADQLVFYINNQYKSNKNVNVRLRQGITVEM*
>TMS91/02324_CYP79D2_C2-6_2
MAMNVSTTATTTASFASTSSMNNTAKILLITLFISIVSTVIKLQKRASYKKASKNFPLPPDSMATHRKHP
*
>TMS91/02324_CYP79D2_C3-2_1
MAMNVSTTATTTASFASTSSMNNTAKILLITLFISIVSTVIKLQKRASYKKASKNFPLPPGPTPWPLIGN
IPEMIRYRPTFRWIHQLMKDMNTDICLIRFGKTNVVPISCPVIAREILKKHDAVFSNRPKILCAKTMSGG
YLTTIVVPYNDQWKKMRKVLTSEIISPARHKWLHDKRAEEADQLVFYINNQYKSNKNVNVRICGKALRWK
CDQKDDV*
>TMS91/02324_CYP79D2_C3-2_2
MAMNVSTTATTTASFASTSSMNNTAKILLITLFISIVSTVIKLQKRASYKKASKNFPLPPGPTPWPLIGN
IPEMIRYRPTFRWIHQLMKDMNTDICLIRFGKTNVVPISCPVIAREILKKHDAVFSNRPKILCAKTMSGG
YLTTIVVPYNDQWKKMRKVLTSEIISPARHKWLHDKRAEEADQLVFYINNQYKSNKNVNVNCGKALRWKC
DQKDDV*
>TMS91/02324_CYP79D2_C3-3_1
MAMNVSTTATTTASFASTSSMNNTAKILLITLFISIVSTVIKLQKRASYKKASKNFPLPPWSDSMATHRK
HP*
>TMS91/02324_CYP79D2_C3-3_2
MAMNVSTTATTTASFASTSSMNNTAKILLITLFISIVSTVIKLQKRASYKKASKNFPLPPVRLHGHSSET
SLK*
>TMS91/02324_CYP79D2_C3-7_1
MAMNVSTTATTTASFASTSSMNNTAKILLITLFISIVSTVIKLQKRASYKKASKNFPLPPGPTPWPLIGN
IPEMIRYRPTFRWIHQLMKDMNTDICLIRFGKTNVVPISCPVIAREILKKHDAVFSNRPKILCAKTMSGG
YLTTIVVPYNDQWKKMRKVLTSEIISPARHKWLHDKRAEEADQLVFYINNQYKSNKNVNVSGKALRWKCD
QKDDV*
>TMS91/02324_CYP79D2_C3-7_2
MAMNVSTTATTTASFASTSSMNNTAKILLITLFISIVSTVIKLQKRASYKKASKNFPLPPWPLIGNIPEM
IRYRPTFRWIHQLMKDMNTDICLIRFGKTNVVPISCPVIAREILKKHDAVFSNRPKILCAKTMSGGYLTT
IVVPYNDQWKKMRKVLTSEIISPARHKWLHDKRAEEADQLVFYINNQYKSNKNVNVRKLRQGITVEM*
