# Supplementary material for: CRISPR-Cas9-mediated knockout of CYP79D1 and CYP79D2 in cassava attenuates toxic cyanogen production
Source: Front Plant Sci. 2023 Mar 17;13:1079254. doi: 10.3389/fpls.2022.1079254 (PMC10064795; doi:10.3389/fpls.2022.1079254)
Supplement: Supplementary file 1 — A Supplementary Materials file including Supplementary Notes 1 and 2, Supplementary Figures 1–12, Supplementary Tables 1–4, and legends for Supplementary Data Files 1–4. [file DataSheet_1.zip › Gomezetal_Supplement_all_final/SupplementaryMaterial_Gomezetal.pdf]

Supplementary Material

Listing of Supplementary Data Files and their legends. 3

Supplementary Note 1 | Vector design, and activity confirmation via geminivirus assay in surrogate model. 4

Supplementary Figure 1 | Cas9 expression system. 5

Supplementary Figure 2 | CRISPR-Cas9 construct activity assay via targeting of surrogate gemini-vector. 6

Supplementary Figure 3 | CRISPR-Cas9 induces indels at *CYP79D1* and *CYP79D2* gRNA target sites in transgenic TME 419 lines. 7

Supplementary Figure 4 | CRISPR-Cas9 induces indels at *CYP79D1* and *CYP79D2* gRNA target sites in transgenic TMS 91/02324 lines. 8

Supplementary Figure 5 | Sanger and Illumina sequence analysis of TME 419 line C3-10 at target site 2B. 9

Supplementary Table 1 | Putative off-target loci in 60444. 10

Supplementary Table 2 | Off-target results for 60444. 11

Supplementary Note 2 | Elimination of cyanogenic glucosides in dual-knockout cassava lines, as measured in *in vitro* plantlets via LC-MS. 12

Supplementary Figure 6 | Cyanogen levels in leaves of edited 60444 *in vitro* plantlets. 13

Supplementary Figure 7 | Cyanogen levels in leaves of edited TME 419 *in vitro* plantlets. 14

Supplementary Figure 8 | Representative 60444 wildtype and genome edited plants and storage roots. 15-16

Supplementary Figure 9 | Dual knockout of *CYP79D1* and *CYP79D2* eliminates cyanide production in accessions 60444, TME 419, and TMS 91/02324. 17

Supplementary Figure 10 | Cyanide levels in roots and leaves of TME 419 *CYP79D1* and *CYP79D2* dual knockout lines. 18

Supplementary Figure 11 | Representative 60444 WT and dual knockout plantlets grown in nutrient- and nitrogen-limited media. 19

Supplementary Table 3 | Media composition for experiment shown in Supplementary Figure 11. 20

Supplementary Figure 12 | Tissue-specific transcript expression of *CYP79D1* and *CYP79D2*. . . . 21

Supplementary Table 4 | PCR primers used in this work. 22-24

References. 25

Supplementary Data Files

Supplementary Data File 1 | Genotypes of CRISPR-Cas9-edited 60444, TME 419, and TMS 91/02324 lines. Genotypes at *CYP79D1* and *CYP79D2* target sites identified by category: wildtype, homozygous, bi-allelic, heterozygous, complex (more than two sequences, indicating mosaicism or chimerism), and number of base pairs inserted (i) and/or deleted (d) for each allele. For bi-allelic genotypes with indels of equivalent size but different base pairs, mutations are distinguished by letters "a" and "b". Deletions that span both target sites are underlined. S (Sanger) or I (Illumina) denotes the type of sequencing applied. Lines originated from the Donald Danforth Plant Science Center (DDPSC) or the Innovative Genomics Institute (IGI). WT, wildtype; N.D., not determined.

Supplementary Data File 2 | Predicted CYP79D amino acid sequences from selected 60444, TME 419, and TMS 91/02324 lines. Amino acid sequences in FASTA format are based on AM560-2 reference assembly v8.1 (Bredeson et al., 2021). Sequence names are presented in order as accession, gene, line, and, in the case of bi-allelic and heterozygous lines, allele number 1 or 2.

Supplementary Data File 3 | LC-MS, picrate, and height readings. LC-MS concentration values reported as <LLOQ (below the lower limit of quantification) were treated as 0 µM. In picrate worksheets, horizontal dotted lines delineate samples of a given line that were analyzed in separate waves of analysis. Multiple stem heights from a single plant are split by comma.

Supplementary Data File 4 | Python notebook for Figures 2 and 3 and Supplementary Figure 9. Code used for generating cyanide content box and whisker plots, and one-to-one group rank sum comparisons. All data used to generate the plots were gathered from picrate paper assays.

Supplementary Notes, Figures, and Tables

Supplementary Note 1 | Vector design, and activity confirmation via geminivirus assay in surrogate model.

We targeted two sites per gene to induce gene disruption by frameshift mutation at either site and/or excision of a substantial portion of the gene (**Figure 1B**). Multiplex editing capability of the CRISPR-Cas9 system was boosted by employing the tRNA-processing system for gRNA expression (Xie et al., 2015).

Target regions of *CYP79D1* and *CYP79D2* were cloned into a plasmid encoding a geminivirus replicon (**Supplementary Figure 2A**). Following *Agrobacterium*-mediated co-delivery and expression of these gemini-vectors and our CRISPR constructs in leaves (**Supplementary Figure 2B**), simultaneous cleavage and excision of the DNA between the two target sites by active Cas9 and gRNAs resulted in shorter target region amplicons (**Supplementary Figure 2C,D**), confirming that the CRISPR constructs were functional.

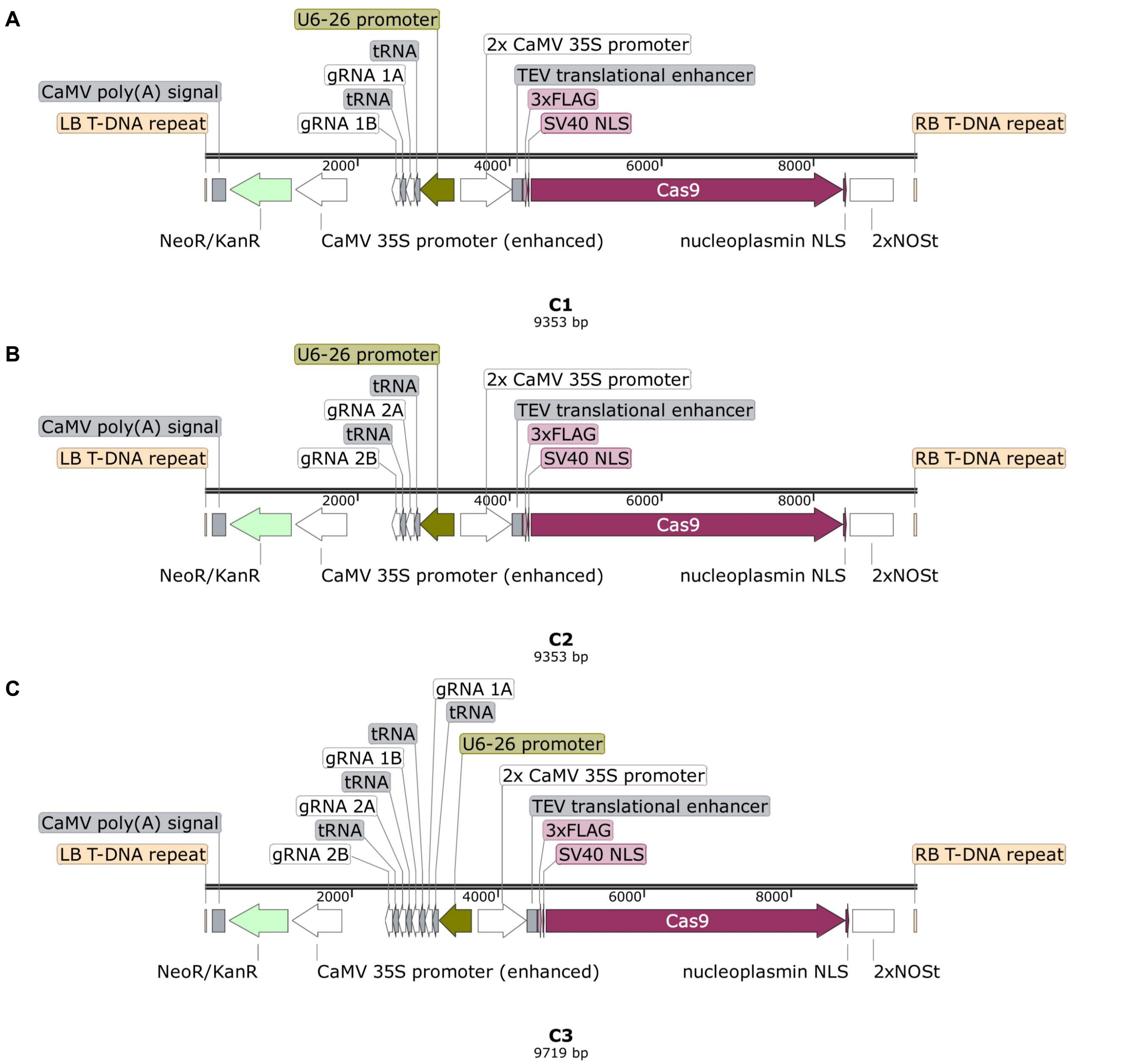

Supplementary Figure 1. Cas9 expression system. 2x35S promoter drives expression of human codon optimized Cas9, preceded by TEV translational enhancer. *Arabidopsis thaliana* U6-26 promoter drives expression of polycistronic tRNA-gRNAs. **(A)** C1 T-DNA, coding for two gRNAs targeting *CYP79D1*. **(B)** C2 T-DNA, coding for two gRNAs targeting *CYP79D2.* **(C)** C3 T-DNA, including four total gRNAs, two each targeting *CYP79D1* and *CYP79D2*. LB, left border; NeoR/KanR, Neomycin/Kanamycin resistance; T-DNA, transfer DNA; gRNA, guide RNA; tRNA, transfer RNA; CaMV, Cauliflower Mosaic Virus; TEV, Tobacco Etch Virus; SV40, Simian Virus 40; NLS, nuclear localization sequence; NOSt, nopaline synthase terminator; RB, right border. Maps created with SnapGene.

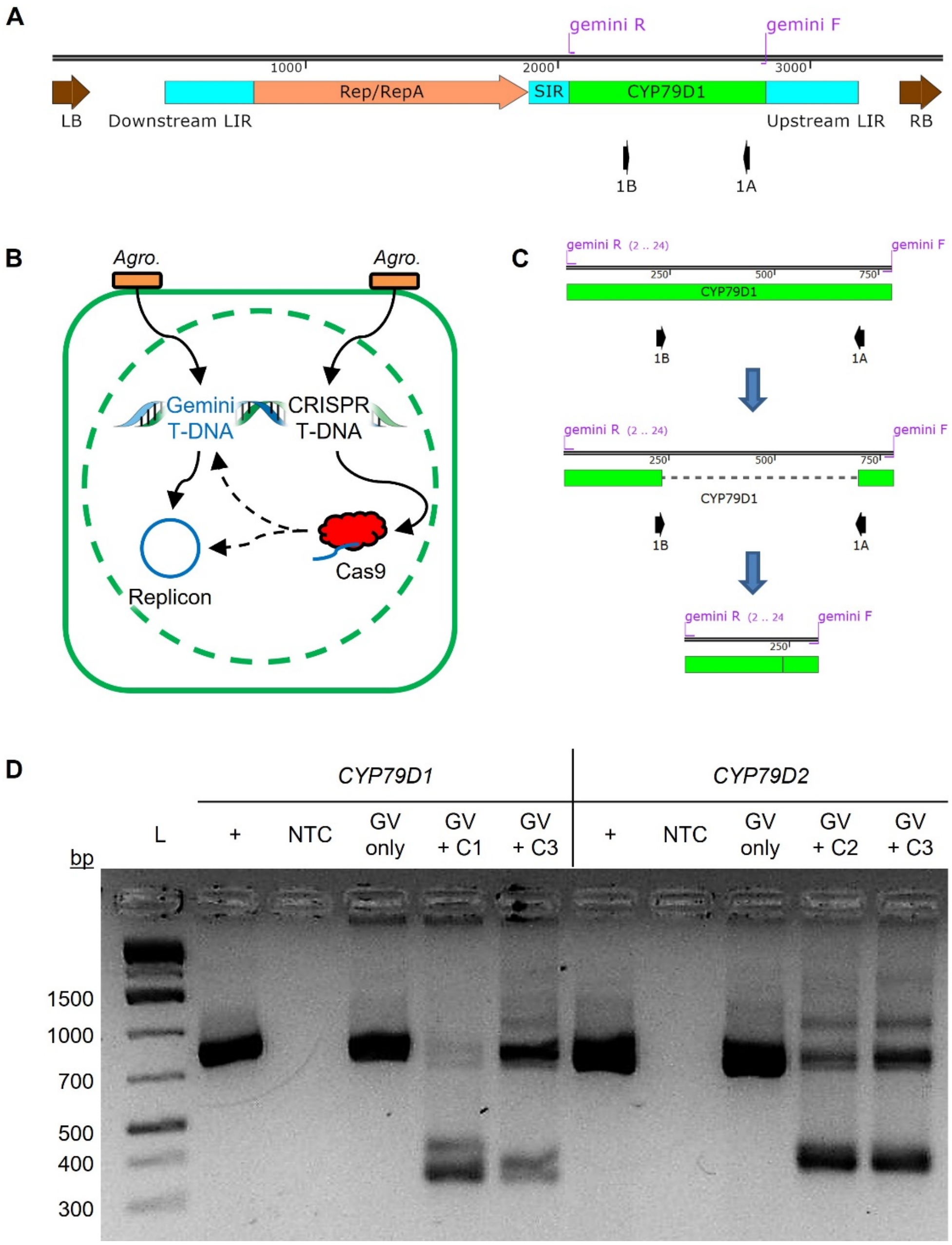

Supplementary Figure 2. CRISPR-Cas9 construct activity assay via targeting of surrogate gemini-vector. **(A)** A 781-bp fragment of *CYP79D1* was cloned within the replication sequences essential for geminiviral replicon synthesis. CRISPR-Cas9 target sites 1A and 1B are within this *CYP79D1* fragment. The replication initiator protein (Rep) initiates rolling-circle replication by binding to the large intergenic region (LIR). Replicons will include the *CYP79D1* fragment. Primer sequences for the amplification of the *CYP79D1* fragment are shown as “gemini F" and “gemini R.” **(B)** The assembled gemini-vector and CRISPR-Cas9 vector encoding the respective gRNA are co-agroinfiltrated into *Nicotiana benthamiana* leaf tissue (nuclear membrane shown as dashed green line). Following expression of the CRISPR-Cas9 system and replicon synthesis, Cas9-gRNA complexes may target either the gemini-vector T-DNA or replicons themselves (paths shown as dashed black lines). Mutagenesis of CRISPR-Cas9 target sites is proliferated in subsequently generated replicons. **(C)** Simultaneous CRISPR-Cas9 mediated cutting of the target sites can cause excision of the intervening sequence. Amplification by primers “gemini F” and “gemini R” now yields a 361-bp fragment. The same approach shown in **a**–**c** was taken with *CYP79D2*, using a 784-bp fragment encompassing target sites 2A and 2B. **(D)** DNA is extracted from leaves infiltrated with the gemini-vector and with/without the respective CRISPR-Cas9 construct (C1, C2, C3). Gel electrophoresis of PCR amplicons shows band sizes of wholly intact and excised DNA fragments. Wholly intact DNA fragments are expected to be 781 bp whereas excised DNA fragments are expected to be 361 bp. +, gemini-vector carrying intact *CYP79D* fragment; NTC, no template control; GV, gemini-vector. Maps created with SnapGene.

A

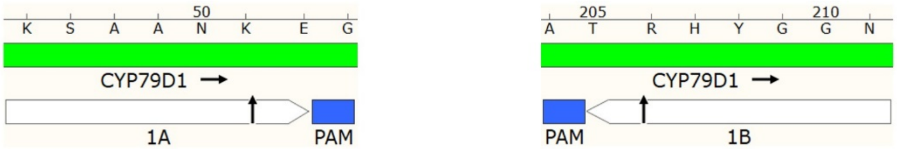

Line	1A sequence	Frame shift	1B sequence	Frame shift
WT	AAGAGTGCTGCTAACAA GGAAGG	×	CCACCA GGCATTACGGCGGGAAT	×
C1-2	AAGAGTGCTGCTAACAATGGAAGG	√	CCACCA GGCATTACGGCGGGAAT<br>CCACCA -GCATTACGGCGGGAAT	×<br>√
C1-9	AAGAGTGCTGCTAACAAAGGAAGG	√	CCA--- ----TTACGGCGGGAAT<br>CCACCAAGGCATTACGGCGGGAAT	√<br>√
C1-10	AAGAGTGCTGCTAACAAAGGAAGG<br>AAGAGTGCTGCTAA--A GGAAGG	√<br>√	CCACCAGGGCATTACGGCGGGAAT<br>CCACCA -GCATTACGGCGGGAAT	√<br>√
C3-4	AAGAGTGCTGCTAAC-- ------...<br>AAGAGTGCTGCTAAC-- --AAGG	 √	...------ -GCATTACGGCGGGAAT<br>CCACCA GGCATTACGGCGGGAAT	√<br>×
C3-44B	AAGAGTGCTGCTA---- ----GG	√	CCACCA G-----ACGGCGGGAAT<br>CCACCA GGCATTACGGCGGGAAT	√<br>×

B

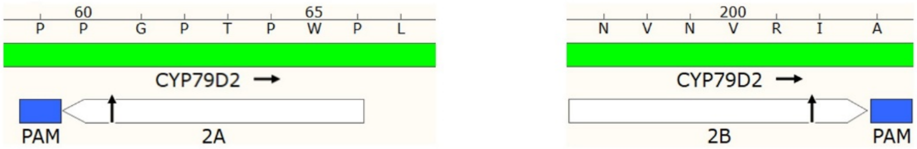

Line	2A sequence	Frame shift	2B sequence	Frame shift
WT	CCCTCCT GGTCCGACTCCATGGCCACTCA	×	GAATGTGAATGTGAGAA TTGCGG	×
C2-6	CCCTCCTAGGTCCGACTCCATGGCCACTCA<br>CCC---- -GTCCGACTCCATGGCCACTCA	√<br>√	GAATGTGAA-------- ------*<br>GAATGTGAATGTGAGAAATTGCGG	√<br>√
C2-7	CCCTCCT ----CGACTCCATGGCCACTCA<br>CCCTCCT -------CTCCATGGCCACTCA	√<br>√	GAATGTGAATGTG---- TTGCGG<br>GAATGTGAATGTGAGAATTTGCGG	√<br>√
C2-10	CCCTCCTAGGTCCGACTCCATGGCCACTCA<br>CCCTCCT -GTCCGACTCCATGGCCACTCA	√<br>√	GAATGTGAATGTGAGA- ----GG<br>GAATGTGAATGTGA--A TTGCGG	√<br>√
C3-4	C------ --------------GCCACTCA<br>CCCTCCT GGTCCGACTCCATGGCCACTCA	√<br>×	GAATGTGAATGTGAGAAATTGCGG<br>GAATGTGAATGTGAGAATTTGCGG	√<br>√
C3-44B	C------ ---------------------A<br>CCCTCCT -GTCCGACTCCATGGCCACTCA	×<br>√	GAATGTGAATGTGAG-- -----G<br>GAATGTGAATGTGAGAATTTGCGG	√<br>√

Supplementary Figure 3. CRISPR-Cas9 induces indels at *CYP79D1* and *CYP79D2* gRNA target sites in transgenic TME 419 lines. Diagrams of the protospacers (white) and protospacer adjacent motifs (PAMs, blue) of *CYP79D1* **(A)** and *CYP79D2* **(B)** gRNA targets are aligned to edited line genotypes. Edited lines are identified by the CRISPR construct with which they were modified (C1, C2, C3), followed by an index number. Black arrow indicates predicted CRISPR-Cas9 cut site. Lengths are to amino acid (top bar) and nucleotide (bottom table) scale. Homozygous genotypes are shown as a single sequence per line. Bi-allelic genotypes are shown as two sequences per line. Multiple mutations to a single allele are row-matched. Insertions are denoted by red, underlined nucleotides. Deletions are denoted by red dashes. Presence of a frameshift mutation at the corresponding target site is denoted by √; absence of a frameshift mutation is denoted by ×. Asterisk denotes a deletion larger than shown: line C2-6 has a 37 bp deletion. Maps created with SnapGene.

A

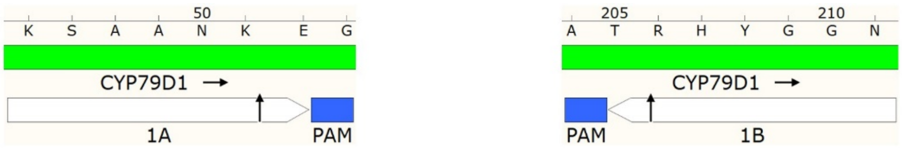

Line	1A sequence	Frame shift	1B sequence	Frame shift
WT	AAGAGTGCTGCTAACAA GGAAGG	×	CCACCAGGCATTACGGCGGGAAT<br>CCACCAGGCATTATGGCGGGAAT	×<br>×
C1-3	AAGAGTGCTGCTAACAATGGAAGG<br>AAGAGTGCTGCTAACAAAGGAAGG	√<br>√	CCACCA---ATTACGGCGGGAAT<br>CCACCAGGCATTATGGCGGGAAT	×<br>×
C1-4	AAGAGTGCTGCTAACAATGGAAGG	√	CCACCAGGCATTACGGCGGGAAT<br>CCACCAGGCATTATGGCGGGAAT	×<br>×
C3-2	AAGAGTGCTGCTAACA- GGAAGG<br>AAGAGTGCTGCTAACAAAGGAAGG	√<br>√	CCACCAGGCATTACGGCGGGAAT<br>CCACCAGGCATTATGGCGGGAAT	×<br>×
C3-3	AAGAGTGCTGCTAACAAAGGAAGG	√	CCACCA-GCATTACGGCGGGAAT<br>CCACCA--CATTATGGCGGGAAT	√<br>√
C3-7	AAGAGTGCTGCTAA--A GGAAGG<br>AAGAGTGCTGCTA---- GGAAGG	√<br>√	CCACCAGGCATTACGGCGGGAAT<br>CCACCAGGCATTATGGCGGGAAT	×<br>×

B

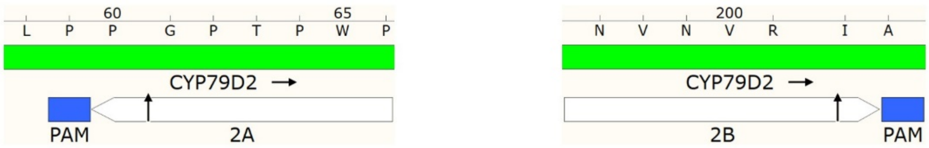

Line	2A sequence	Frame shift	2B sequence	Frame shift
WT	CTCCCTCCT GGTCCGACTCCATGGCC	×	GAATGTGAATGTGAGAA TTGCGG	×
C2-6	C-------- -GTCCGACTCCATGGCC<br>CTCCCTCCT -----GACTCCATGGCC	×<br>√	GAATGTGAATGTGAGA- TTGCGG<br>GAATGTGAATGTGA--- TTGCGG	√<br>×
C3-2	CTCCCTCCT GGTCCGACTCCATGGCC	×	GAATGTGAATGTGAGAAT TTGCGG<br>GAATGTGAATGTGA--A TTGCGG	√<br>√
C3-3	CTCCCTCCTTGGTCCGACTCCATGGCC<br>CTCCCTCCT -GTCCGACTCCATGGCC	√<br>√	GAATGTGAATGTGA--- TTGCGG	×
C3-7	CTCCCTCCT GGTCCGACTCCATGGCC<br>CTCCCTCC- -----------ATGGCC	×<br>×	GAATGTGAATGTGAG-- ---CGG<br>GAATGTGAATGTGAGAAAATTGCGG	√<br>√

Supplementary Figure 4. CRISPR-Cas9 induces indels at *CYP79D1* and *CYP79D2* gRNA target sites in transgenic TMS 91/02324 lines. Diagrams of the protospacers (white) and protospacer adjacent motifs (PAMs, blue) of *CYP79D1* **(A)** and *CYP79D2* **(B)** gRNA targets are aligned to edited line genotypes. Edited lines are identified by the CRISPR construct with which they were modified (C1, C2, C3), followed by an index number. Black arrow indicates predicted CRISPR-Cas9 cut site. Lengths are to amino acid (top bar) and nucleotide (bottom table) scale. Homozygous genotypes are shown as a single sequence per line. Bi-allelic genotypes are shown as two sequences per line. Multiple mutations to a single allele are row-matched. Insertions are denoted by red, underlined nucleotides. Deletions are denoted by red dashes. Presence of a frameshift mutation at the corresponding target site is denoted by √; absence of a frameshift mutation is denoted by ×. Maps created with SnapGene.

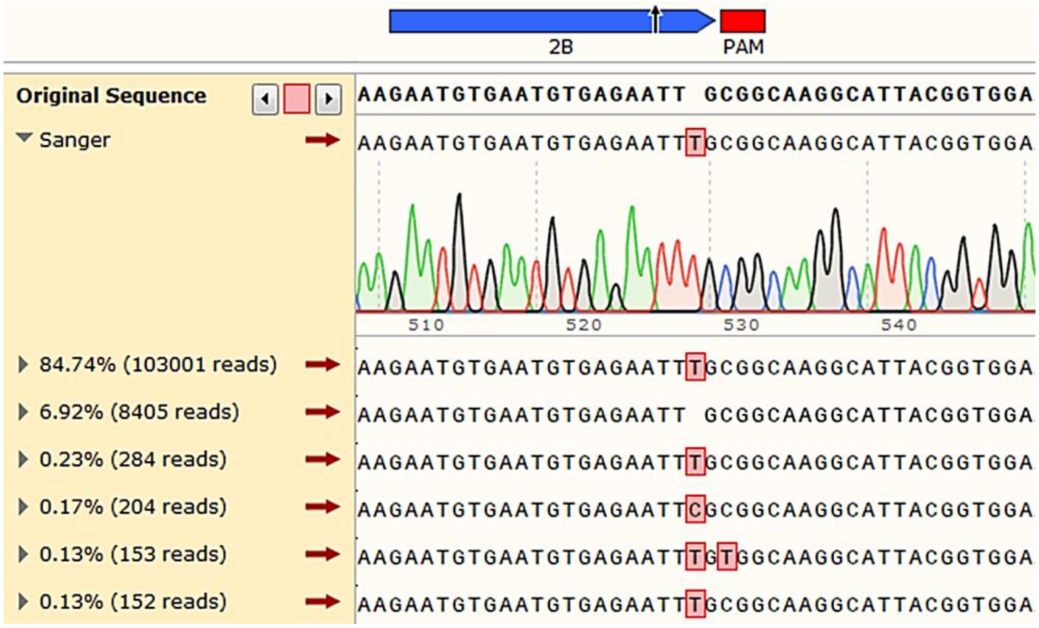

Supplementary Figure 5. Sanger and Illumina sequence analysis of TME 419 line C3-10 at target site 2B. Diagram of the protospacer (blue) and protospacer adjacent motif (PAM, red) of the target is aligned to the nucleotide sequence. Black arrow indicates predicted CRISPR-Cas9 cut site. Original sequence (wildtype) is shown in bold letters. Sanger sequence is shown with chromatogram analysis; unique Illumina sequences are shown below. Mismatches are highlighted in red boxes. Some distinguishing mismatches between the read sets are outside of the figure window. Map created with SnapGene.

Supplementary Table 1. Putative off-target loci in 60444. Highest ranking off-targets identified by CasOT utilizing the 60444 genome assembly (Gomez et al., 2019). Lower case letters in the Sequence column identify mismatches with the target sequence. Non-seed and seed regions separated by underscore. Spacer and PAM separated by hyphen. Locations and gene identifiers provided are for cassava AM560-2 v6.1 reference genome.

Gene	Target	#	Sequence	Number of mismatches (Non-seed, seed)	V6.1 Location	Region	Gene Identifier
CYP79D1	1A	1	tAaAaaca_TGCTAACAAGGA-TGG	6, 0	Chromosome04: 3401297..3401319	intergenic region	-
		2	AgagtaGt_TGCTAACAAGGA-TGG	6, 0	Chromosome08: 1654375..1654397	intron	Manes.08G017500
	1B	1	AgTggCcC_CGTgATGCCTGG-TGG	4, 1	Chromosome05: 4248805..4248827	intergenic region	-
		2	ATatCgGa_CGTgATGCCTGG-CGG	4, 1	Chromosome08: 4989884..4989906	intergenic region	-
CYP79D2	2A	1	GaCttaGG_AGTCtGACCAGG-AGG	4, 1	Chromosome02: 18109801..18109827	intergenic region	-
		2	GGggAatG_AGTCGGACgAGG-TGG	4, 1	Chromosome04: 17588060..17588082	exon	Manes.04G064300
		3	ttCttTGG_AGTCGGAgCAGG-AGG	4, 1	Chromosome15: 10501754..10501776	intergenic region	-
	2B	1	cccTaaGA_ATGTGAGAATTG-AGG	5, 0	Chromosome03: 17132076..17132098	intron	Manes.03G098600
		2	GgtTtgtA_ATGTGAGAATTG-AGG	5, 0	Chromosome15: 9415632..9415654	intergenic region	-

Supplementary Table 2. Off-target results for 60444. Highest ranked potential off-targets (O.T.) were examined by Sanger sequence analysis for conservation of the wildtype (WT) sequence. Check marks indicate sequences from the WT line matched the 60444 assembly. Edited lines are identified by the CRISPR construct with which they were modified (C1, C2, C3), followed by an index number (e.g., 142A). Corresponding loci in edited lines that matched the WT sequence are marked as WT. Loci that could not be amplified and sequenced are marked as not determined (N.D.).

Gene	Target	O.T.	WT	C1-4	C1-6	C2-4	C2-2	C3-2	C3-142A	C3-145C
CYP79D1	1A	1	✓	WT	WT			WT	WT	WT
		2	✓	WT	WT			WT	WT	WT
	1B	1	✓	WT	WT			WT	WT	WT
		2	✓	WT	WT			WT	WT	WT
CYP79D2	2A	1	✓			N.D.	N.D.	N.D.	N.D.	N.D.
		2	✓			WT	WT	WT	WT	WT
		3	✓			WT	WT	WT	WT	WT
	2B	1	✓			WT	WT	WT	WT	WT
		2	✓			WT	WT	WT	WT	WT

Supplementary Note 2 | Elimination of cyanogenic glucosides in dual-knockout cassava lines, as measured in *in vitro* plantlets via LC-MS.

We measured levels of linamarin and lotaustralin in leaves of edited 60444 and TME 419 *in vitro* plantlets using liquid chromatography-mass spectrometry (LC-MS), with age-matched wildtype *in vitro* plantlets as positive controls (**Methods, Supplementary Figures 6,7**). A single leaf sample was taken from each of one to three plants per line, and an average value for each plant calculated from three technical replicates. No linamarin or lotaustralin was detected in negative (no tissue) controls.

Linamarin was not detected in dual knockout lines derived from either 60444 or TME 419 (**Supplementary Figure 6A**, **Supplementary Figure 7A**). Linamarin values of a 60444-derived *CYP79D1* knockout line (0.12–0.17 g per kg fresh weight [g/kgfw]) fell within the range of wildtype values (0.11–0.40 g/kgfw). Linamarin values for three TME 419-derived *CYP79D1* knockout lines were more variable, ranging from undetectable to 0.47 g/kgfw, while wildtype ranged from 0.15–0.23 g/kgfw. *CYP79D2* knockouts, however, in both accessions displayed linamarin levels consistently much lower than wildtype: highest at 0.03 g/kgfw in 60444 and 0.10 in TME 419, with two out of three TME 419 lines at or below 0.01 g/kgfw. A low level of linamarin was detected in all three assayed plantlets of TME 419 line C3-10 (0.003–0.020 g/kgfw). This line had appeared from Sanger sequencing to be a double knockout mutant; Illumina amplicon sequencing, however, showed 6.9% wildtype reads at site 2B (**Supplementary Figure 5**). We interpreted this result as revealing this line is complex, with some cells having an intact *CYP79D2* gene, and we did not consider this line further.

Lotaustralin measurements from *in vitro* plantlets generally followed the trend seen for linamarin, except at much lower levels (**Supplementary Figure 6B**, **Supplementary Figure 7B**). Wildtype values reached 0.019 g/kgfw in 60444 and 0.007 g/kgfw in TME 419. Low lotaustralin levels were expected, since linamarin is the predominant cyanogen in cassava (Nartey, 1968). Lotaustralin was only detected in one *CYP79D2* knockout plant of one line in each of the two accessions. Extremely small amounts (0.0005 and 0.0007 g/kgfw) of lotaustralin were detected in two 60444 double knockout plants. As no linamarin was detected in any sample from these lines, and no lotaustralin was detected in the other plants from these lines, it is unlikely that these readings reflect true cyanogenesis but may instead be the result of technical contamination.

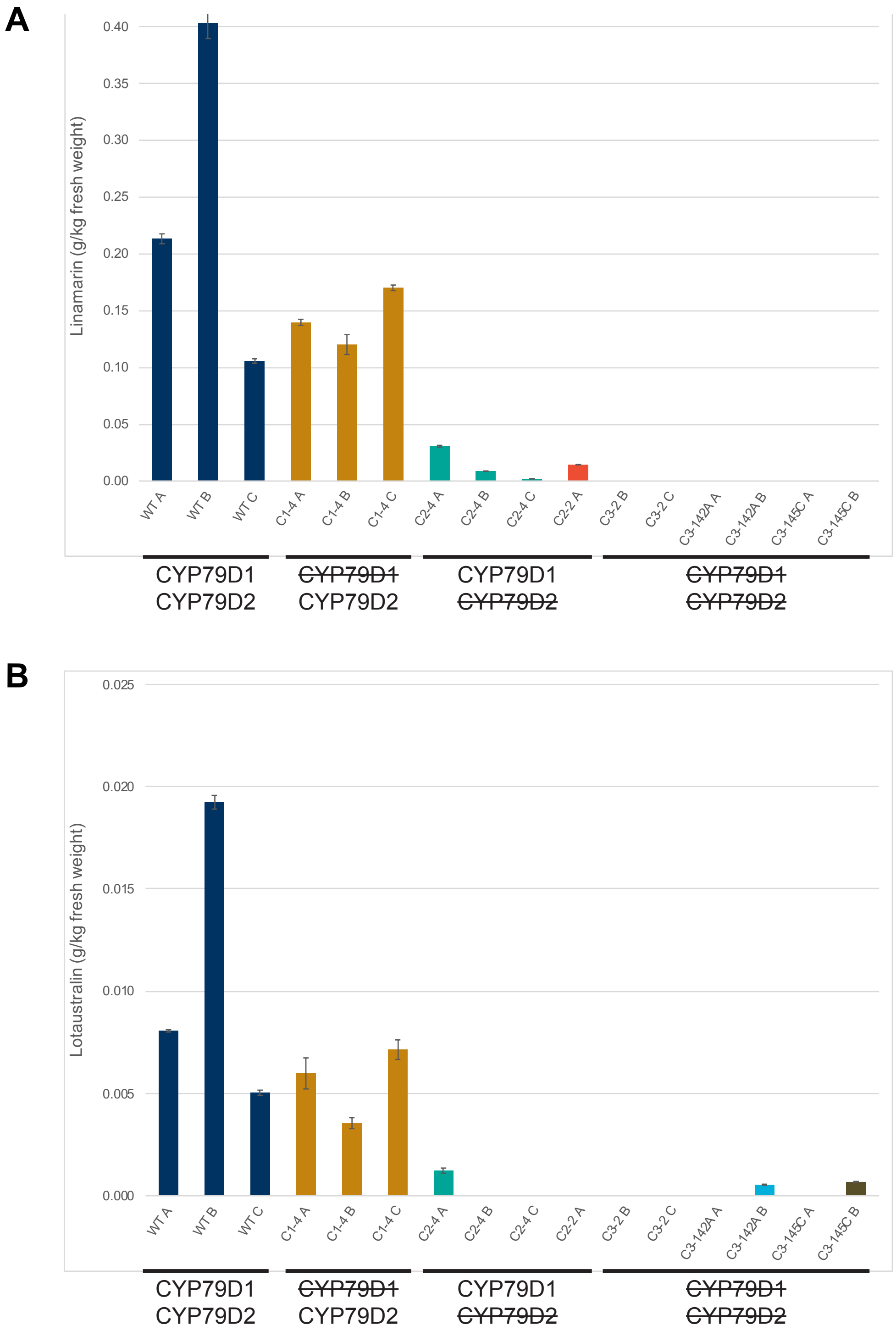

Supplementary Figure 6. Cyanogen levels in leaves of edited 60444 *in vitro* plantlets. **(A,B)** Linamarin **(A)** and lotaustralin **(B)** levels in g/kg fresh weight, as measured via LC-MS. Each bar represents one leaf sample from one plant, as the mean of three technical replicates. Error bars are standard error, calculated from technical replicates. Values below the limit of quantification were treated as 0; standard error was not calculated for these. Line identifiers are followed by a plant identifier (A, B, or C). Bars of the same color are plants of the same line. Edited lines are identified by the CRISPR construct with which they were modified (C1, C2, C3), followed by an index number (e.g., 142A).

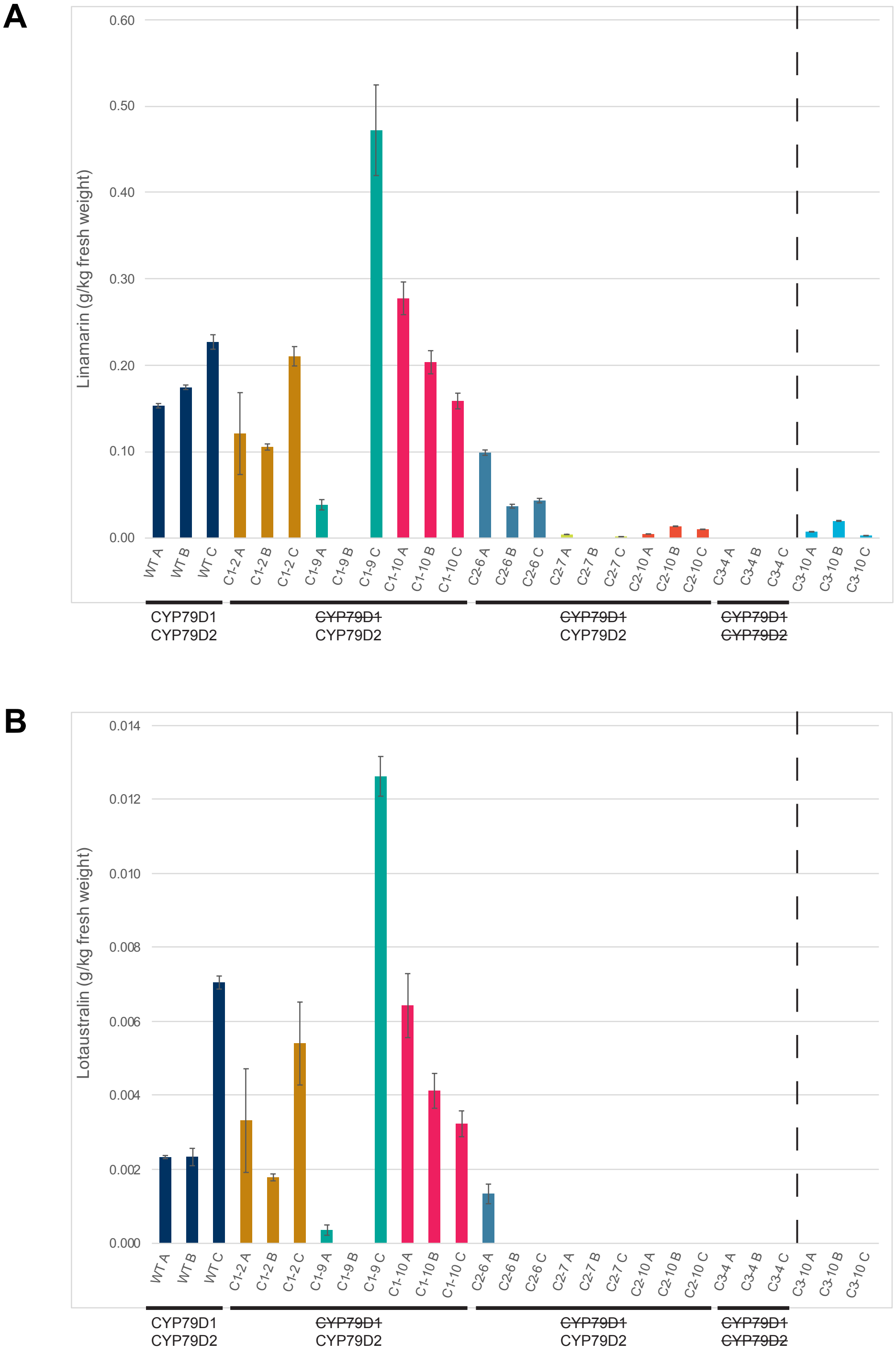

Supplementary Figure 7. Cyanogen levels in leaves of edited TME 419 *in vitro* plantlets. **(A,B)** Linamarin (**A**) and lotaustralin (**B**) levels in g/kg fresh weight, as measured via LC-MS. Each bar represents one leaf sample from one plant, as the mean of three technical replicates. Error bars are standard error, calculated from technical replicates. Values below the limit of quantification were treated as 0; standard error was not calculated for these. Line identifiers are followed by a plant identifier (A, B, or C). Bars of the same color are plants of the same line. Line C3-10 is mosaic for a wildtype *CYP79D2* allele. Edited lines are identified by the CRISPR construct with which they were modified (C1, C2, C3), followed by an index number.

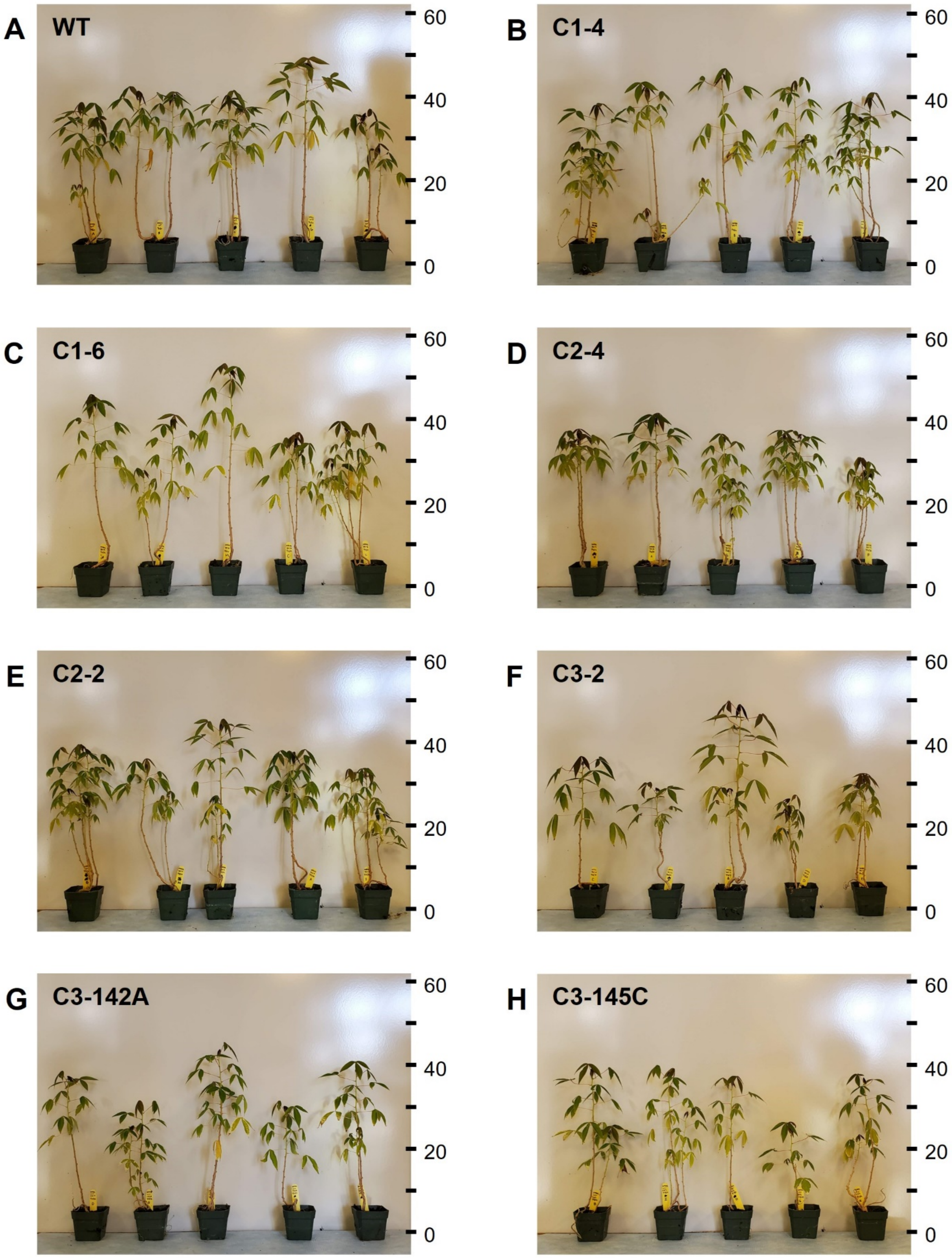
A
WT
B
C1-4
C
C1-6
D
C2-4
E
C2-2
F
C3-2
G
C3-142A
H
C3-145C
60
40
20
0

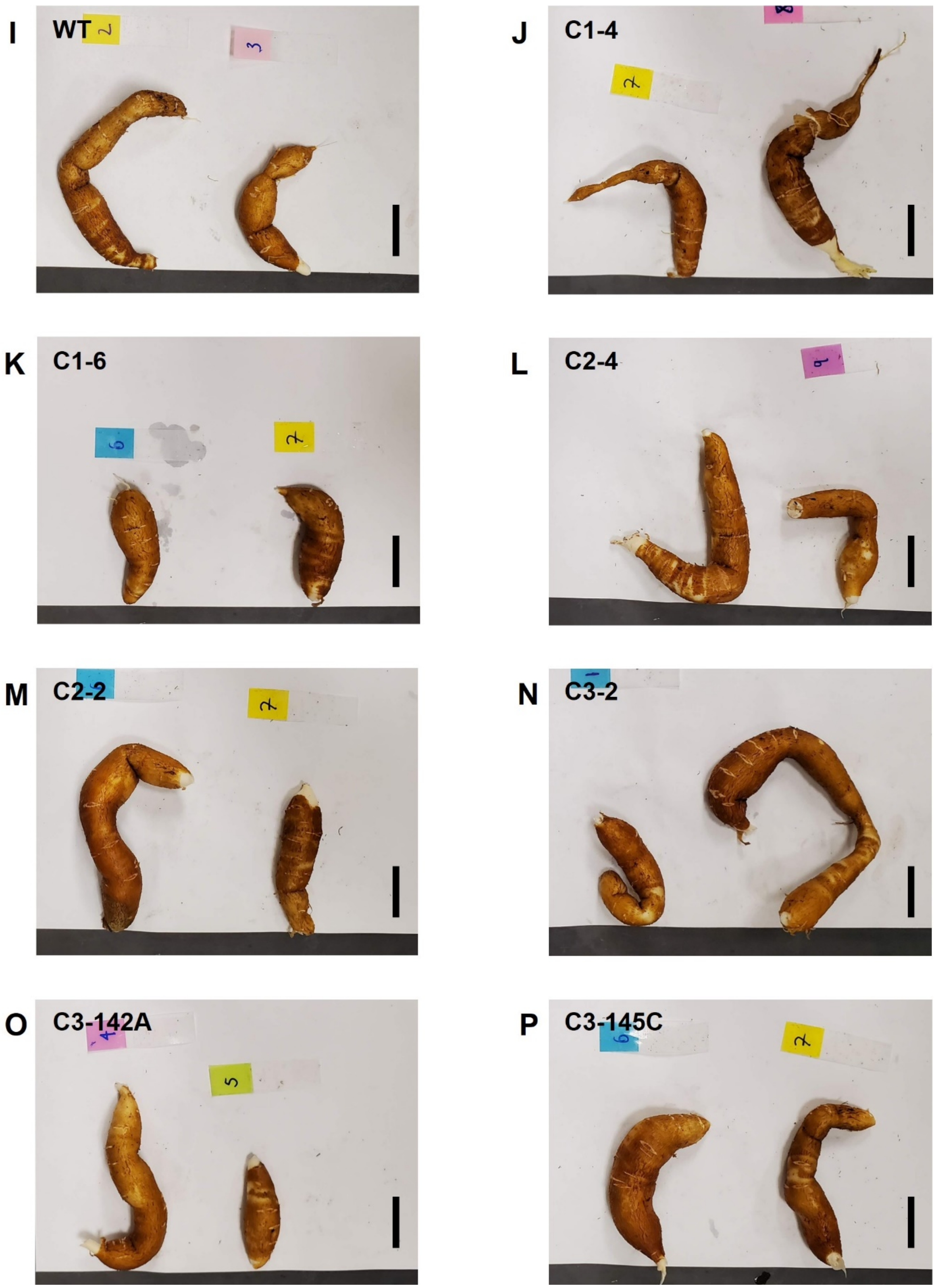

Supplementary Figure 8. Representative 60444 wildtype and genome edited plants and storage roots. **(A–H)** Plants growing in 3-inch pots eight months after transfer to soil. Scale is in centimeters. **(I–P)** Roots harvested from plants grown in 3-inch pots 6.5 months after transfer to soil. Black bar represents 2 cm.

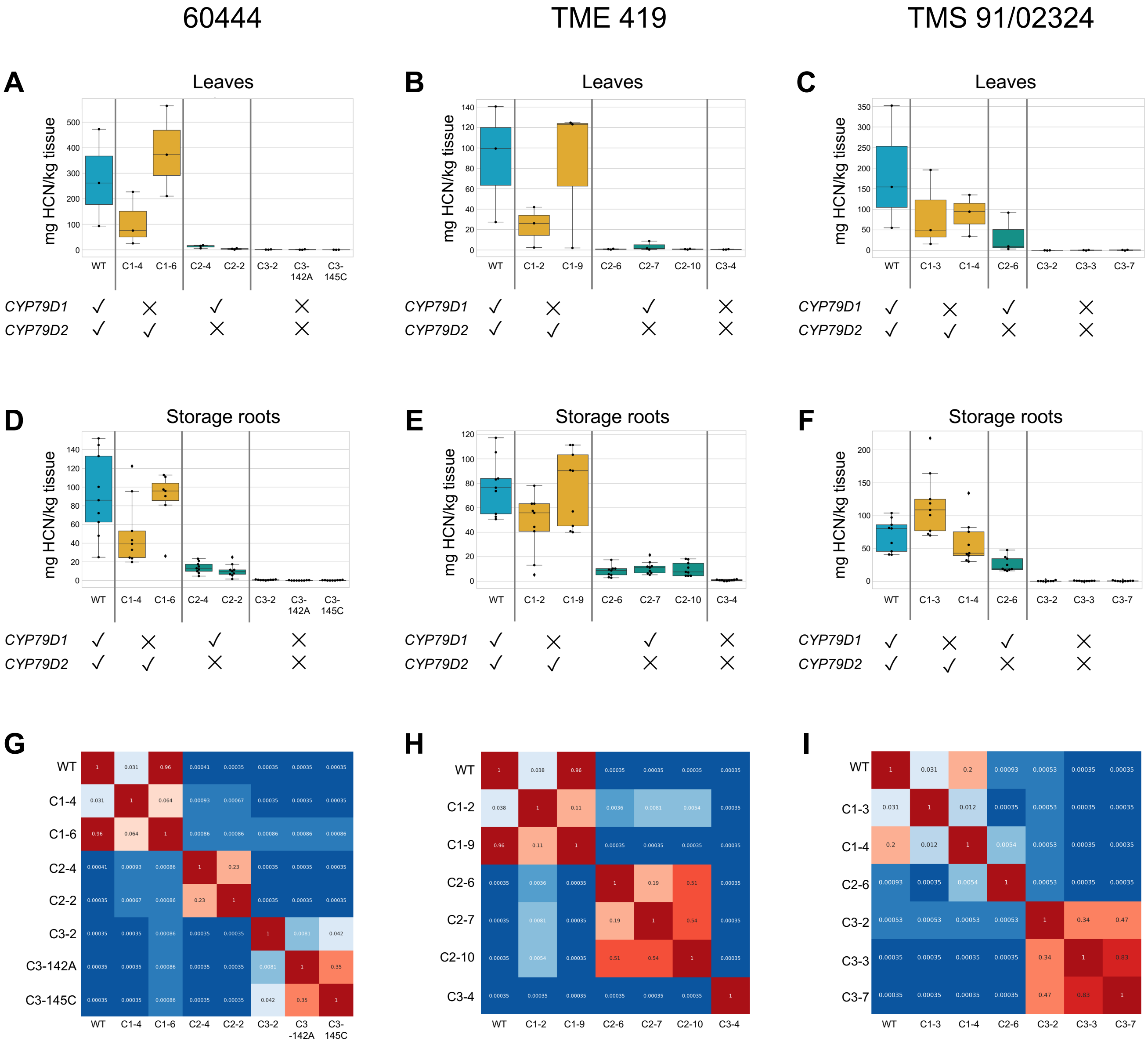

Supplementary Figure 9. Dual knockout of *CYP79D1* and *CYP79D2* eliminates cyanide production in accessions 60444, TME 419, and TMS 91/02324. Assays were conducted 6.5, 8, and 7.5 and months, respectively, after plants were transferred to soil. **(A–F)** Box and whisker plots of cyanide values in leaves **(A–C)** and storage roots **(D–F)** in mg HCN per kg tissue, as detected by picrate assay. Black dots are biological replicates. The median, and lower (25th percentile) and upper (75th percentile) quartiles are indicated. Whiskers define the minimum and maximum regions of the data; data points outside of these are outliers. **(G–I)** Wilcoxon Rank Sum p-values calculated from pairwise comparisons between lines of root cyanide content. Values less than 0.05, indicating the distributions of the values are statistically different between the two lines, are colored in shades of blue. Values greater than 0.05 are colored in shades of red. WT, wildtype. Edited lines are identified by the CRISPR construct with which they were modified (C1, C2, C3), followed by an index number (e.g., 142A).

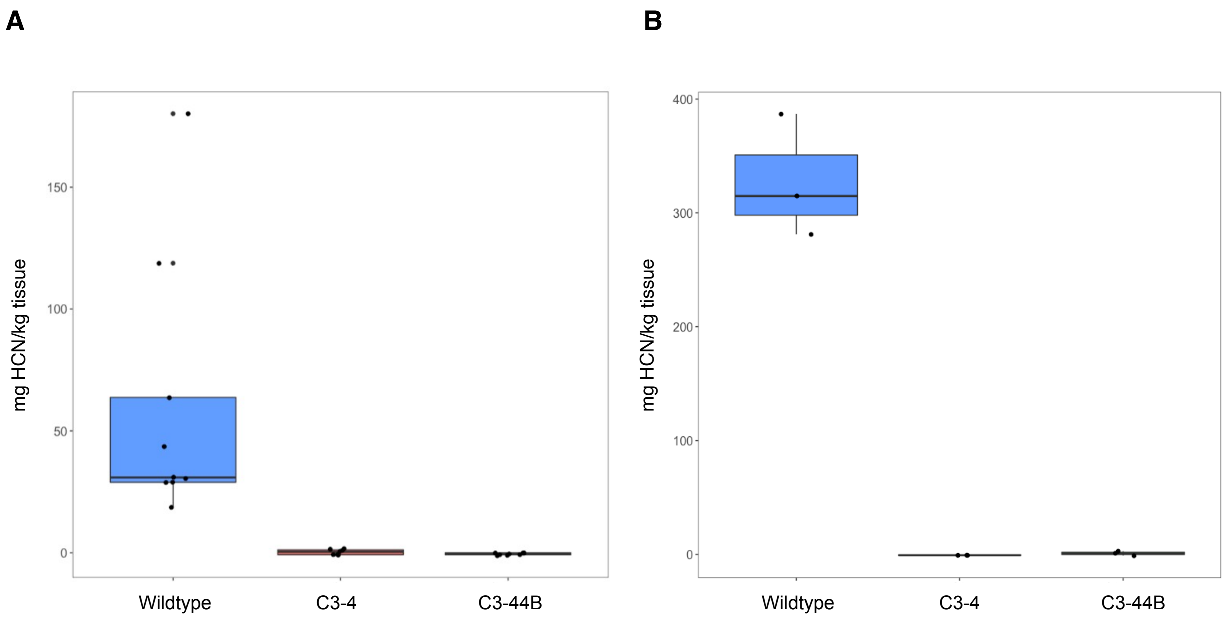

Supplementary Figure 10. Cyanide levels in roots and leaves of TME 419 *CYP79D1* and *CYP79D2* dual knockout lines. Box and whisker plots of cyanide values in roots **(A)** and leaves **(B)** in mg HCN per kg tissue, as detected by picrate assay. Individual samples are represented as black dots. The median, and lower (25th percentile) and upper (75th percentile) quartiles are indicated. Whiskers define the minimum and maximum regions of the data; data points that fall outside this range are outliers. Wilcoxon Rank Sum p-values were calculated from pairwise comparisons between lines of cyanide content. Values less than 0.05, indicating the distributions of the values are statistically different between each group, are indicated by letters. WT, wildtype. Edited lines are identified by the CRISPR construct with which they were modified (C3), followed by an index number (e.g., 44B).

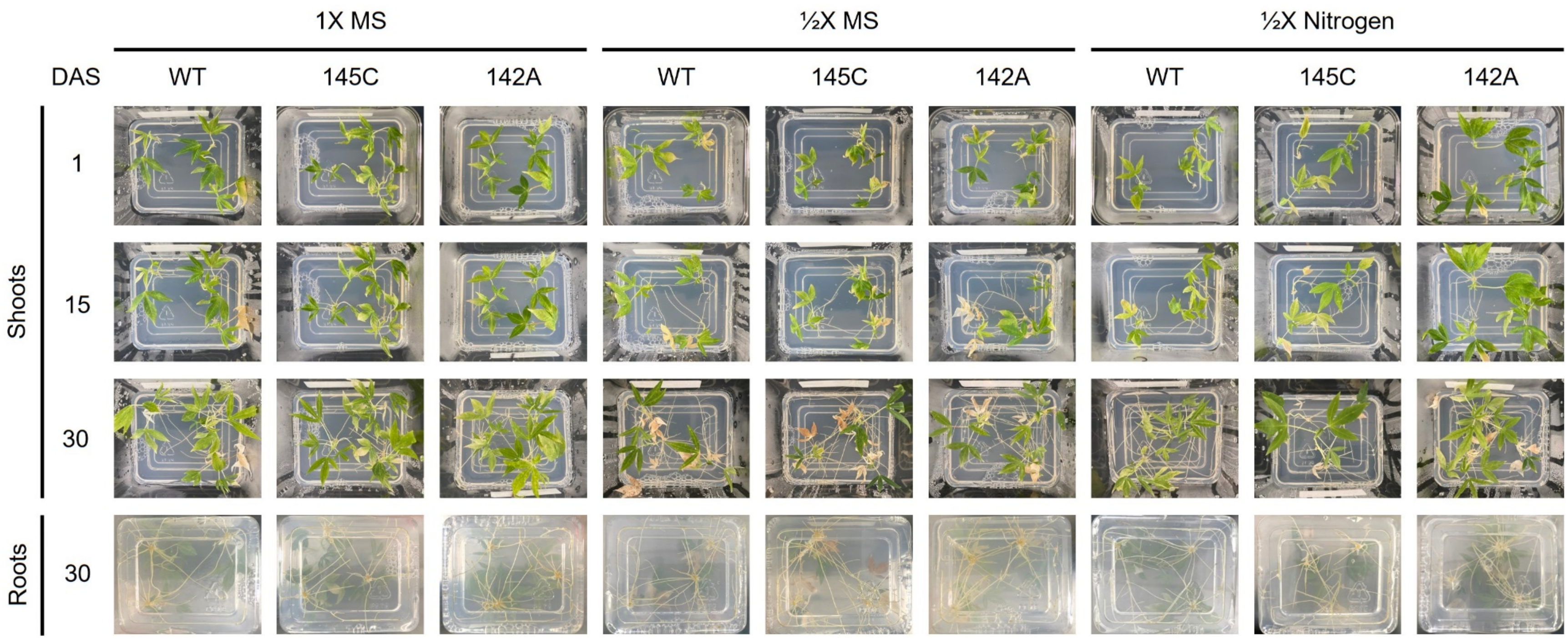

Supplementary Figure 11. Representative 60444 WT and dual knockout plantlets grown in nutrient- and nitrogen-limited media. *In vitro* plants were staked onto MS media with 1X, ½X MS and ½X nitrogen. Plantlets were grown in PhytaTray II containers, the bases of which are 9 cm x 7.5 cm. Shoot and root systems are shown from above and below, respectively, at 1, 15, and 30 days after staking (DAS).

Supplementary Table 3. Media composition for experiment shown in Supplementary Figure 11.

Category	Component	1X MS (mg/L)	½X MS (mg/L)	½X Nitrogen (mg/L)
**Macronutrients**	Potassium Nitrate	1900	950	1900
	Ammonium Nitrate	1650	825	825
	Calcium Chloride, Anhydrous	332.2	166.1	332.2
	Magnesium Sulfate, Anhydrous	180.7	90.35	180.7
	Potassium Phosphate, Monobasic	170	85	170
**Micronutrients**	$Na_2EDTA \cdot 2H_2O$	37.26	18.63	37.26
	Ferrous Sulfate$\cdot 7H_2O$	27.8	13.9	27.8
	Manganese Sulfate$\cdot H_2O$	16.9	8.45	16.9
	Zinc Sulfate$\cdot 7H_2O$	8.6	4.3	8.6
	Boric Acid	6.2	3.1	6.2
	Potassium Iodide	0.83	0.415	0.83
	Molybdic Acid (Sodium Salt)$\cdot 2H_2O$	0.25	0.125	0.25
	Cobalt Chloride$\cdot 6H_2O$	0.025	0.0125	0.025
	Cupric Sulfate$\cdot 5H_2O$	0.025	0.0125	0.025
**Vitamins**	myo-Inositol	100	50	100
	Glycine (Free Base)	2	1	2
	Thiamine•HCl	1	0.5	1
	Nicotinic Acid (Free Acid)	0.5	0.25	0.5
	Pyridoxine•HCl	0.5	0.25	0.5

A *CYP79D1* **B** *CYP79D2*

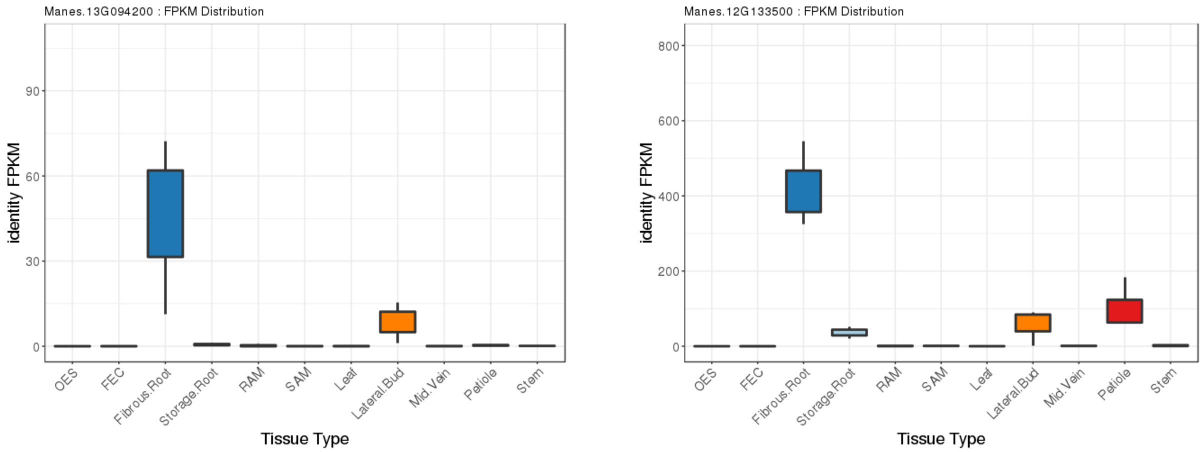

Supplementary Figure 12. Tissue-specific transcript expression of *CYP79D1* and *CYP79D2*. Graphs for CYP79D1 **(A)** and CYP79D2 **(B)** expression were produced using the Cassava Atlas tool (Wilson et al., 2017) (http://shiny.danforthcenter.org/cassava_atlas). African cassava accession TME 204 was sampled for gene expression 3 months after planting. Each box and whisker plot shows gene expression levels across cassava tissues/organs. FPKM, fragments per kilobase of transcript per million; OES, organized embryogenic structures; FEC, friable embryogenic callus; SAM, shoot apical meristem; RAM, root apical meristem.

Supplementary Table 4. PCR primers used in this work.

Name	Sequence	Function
CYP79D-1 TR F	CAACACGGTCAAGATCTTGTTCG	preliminary seq analysis of target region
CYP79D-2 TR F	GAACAATACTGCCAAAATCCTCC	preliminary seq analysis of target region
CYP79D TR R	ATCCCTTATGGTCTTATTTGCAT	preliminary seq analysis of target region
gemini-CYP79D1 TR F	AATTATTCGTACGACCCTCCCAACACGGTCAAGATCTTG	seq analysis and gemini-vector cloning
gemini-CYP79D2 TR F	AATTATTCGTACGACCCTCCGAACAATACTGCCAAAATC	seq analysis and gemini-vector cloning
CYP79D-gemini TR R	CATAAAATAATCATTTTATTCATCCCTTATGGTCTTATTTG	seq analysis and gemini-vector cloning
CYP79D1 g1A-F	TAGGTCTCCTGCTAACAAGGAGTTTAAGAGCTATGC	gRNA assembly into PTG array
CYP79D1 g1A-R	ATGGTCTCAAGCAGCACTCTTTGCACCAGCCGGGAA	gRNA assembly into PTG array
CYP79D1 g1B-F	TAGGTCTCCCGTAATGCCTGGGTTTAAGAGCTATGC	gRNA assembly into PTG array
CYP79D1 g1B-R	ATGGTCTCATACGGCGGGAATTGCACCAGCCGGGAA	gRNA assembly into PTG array
CYP79D2 g2A-F	TAGGTCTCCAGTCGGACCAGGGTTTAAGAGCTATGC	gRNA assembly into PTG array
CYP79D2 g2A-R	ATGGTCTCAGACTCCATGGCCTGCACCAGCCGGGAA	gRNA assembly into PTG array
CYP79D2 g2B-F	TAGGTCTCCATGTGAGAATTGGTTTAAGAGCTATGC	gRNA assembly into PTG array
CYP79D2 g2B-R	ATGGTCTCAACATTCACATTCTGCACCAGCCGGGAA	gRNA assembly into PTG array
Ampseq-CYP79D1-g1A F	GCTCTTCCGATCTCCACCATCGGTTTACTTAACG	MiSeq analysis
Ampseq-CYP79D1-g1A R	GCTCTTCCGATCTAACAAAGTTAGTTCTTCCAAAACG	MiSeq analysis
Ampseq-CYP79D1-g1B F	GCTCTTCCGATCTTCTTAACCTCAGAGATCATTTCTCC	MiSeq analysis
Ampseq-CYP79D1-g1B R	GCTCTTCCGATCTGAAAGGCAAGAAATCTGATATGC	MiSeq analysis
Ampseq-CYP79D2-g2A F	GCTCTTCCGATCTCGTCCTCCATGAACAATACTGC	MiSeq analysis
Ampseq-CYP79D2-g2A R	GCTCTTCCGATCTATTTCACGAGCAATGACAGG	MiSeq analysis
Ampseq-CYP79D2-g2B F	GCTCTTCCGATCTTCCTAACTTCAGAGATCATTTCTCC	MiSeq analysis
Ampseq-CYP79D2-g2B R	GCTCTTCCGATCTAAAAGGCAAGTAATCAGAGATGC	MiSeq analysis

iSeq - CYP79D1 g1A F	GCTCTTCCGATCTCACCTCCTTCGCCTCCTC	iSeq analysis
iSeq - CYP79D1 g1A R	GCTCTTCCGATCTTGAGTTGGTGAATCCACCG	iSeq analysis
iSeq - CYP79D1 g1B F	GCTCTTCCGATCTGCTAGACACAAATGGCTCCATG	iSeq analysis
iSeq - CYP79D1 g1B R	GCTCTTCCGATCTACGGCATCAATGTGCTCG	iSeq analysis
iSeq - CYP79D2 g2A F	GCTCTTCCGATCTTACTGCCAAAATCCTCCTTATCAC	iSeq analysis
iSeq - CYP79D2 g2A R	GCTCTTCCGATCTTCAGACAAATATCGGTGTTCATGTC	iSeq analysis
iSeq - CYP79D2 g2B F	GCTCTTCCGATCTATTTCTCCAGCTAGGCACAAATG	iSeq analysis
iSeq - CYP79D2 g2B R	GCTCTTCCGATCTACGTGCATGATTTCTTCAGG	iSeq analysis
1A OT1 F	GCAAGTTGCATGGAAGTCTCTC	off target analysis
1A OT1 R	TCCTTCTTACATGCTTCTCAAGG	off target analysis
1A OT2 F	AGGACAGAAATGGAATGATGC	off target analysis
1A OT2 R	TATCCAAGAGGGGCTCGTAG	off target analysis
1B OT1 F	TGCAAAACATGTCAGCTCAAC	off target analysis
1B OT1 R	TCACCACATATACTGCCTTTGC	off target analysis
1B OT2 F	GCCGGGTATTACATCCTTCC	off target analysis
1B OT2 R	ATGCCAAGTCACAAGGTGAG	off target analysis
2A OT1 F	ATGCCTCTCCGCTATAGGAC	off target analysis
2A OT1 R	AGTTTGATCATGCTAAATGAAGG	off target analysis
2A OT2 F	TTGATATCTATTATTCGTTTTCTGGAC	off target analysis
2A OT2 R	TCATCAATTGCAAGGCTCTTC	off target analysis
2A OT3 F	AAATAGTCATTTCGTCTATTTTTGC	off target analysis
2A OT3 R	ACGAAATAGTCCTTCCTCATCG	off target analysis
2B OT1 F	GAATTAGGGAGGAAATGACAAAAG	off target analysis
2B OT1 R	CACCATTTCTTCTTGCAAAGC	off target analysis

2B OT2 F	ATTTTCTTTTTCAATTCTAACTTCAAC	off target analysis
2B OT2 R	GGCACATGCGACTTCTGTG	off target analysis
CYP79D1 5UTR F	GCGATATCCCTGGATTTG	cDNA analysis
CYP79D1 3UTR R	ATTAAAGGACGTTCTAAGAAC	cDNA analysis
CYP79D2 5UTR F	GTATGGTCTTGGTCATAGC	cDNA analysis
CYP79D2 3UTR R	CTAACAACTCACATTCATCC	cDNA analysis

References

Bredeson, J. V., Shu, S., Berkoff, K., Lyons, J. B., Caccamo, M., Santos, B., et al. (2021). An improved reference assembly for cassava (*Manihot esculenta* Crantz). https://phytozome-next.jgi.doe.gov/info/Mesculenta_v8_1; NCBI genome ID GCA_001659605.2. Available at: https://phytozome-next.jgi.doe.gov/info/Mesculenta_v8_1; NCBI genome ID GCA_001659605.2.

Gomez, M. A., Lin, Z. D., Moll, T., Chauhan, R. D., Hayden, L., Renninger, K., et al. (2019). Simultaneous CRISPR/Cas9-mediated editing of cassava *eIF4E* isoforms *nCBP-1* and *nCBP-2* reduces cassava brown streak disease symptom severity and incidence. *Plant Biotechnol. J.* 17, 421–434.

Nartey, F. (1968). Studies on cassava, Manihot utilissima Pohl—I. Cyanogenesis: The biosynthesis of linamarin and lotaustralin in etiolated seedlings. *Phytochemistry* 7, 1307–1312.

Wilson, M. C., Mutka, A. M., Hummel, A. W., Berry, J., Chauhan, R. D., Vijayaraghavan, A., et al. (2017). Gene expression atlas for the food security crop cassava. *New Phytol.* 213, 1632–1641.

Xie, K., Minkenberg, B., and Yang, Y. (2015). Boosting CRISPR/Cas9 multiplex editing capability with the endogenous tRNA-processing system. *Proc. Natl. Acad. Sci. U. S. A.* 112, 3570–3575.
